# Clinical, neurophysiological and neurochemical effects of non-invasive electrical brain stimulation in fibromyalgia syndrome—a systematic review and meta-analysis

**DOI:** 10.3389/fpain.2025.1593746

**Published:** 2025-08-01

**Authors:** Christine Winterholler, Maria Helena Coura, Pedro Montoya

**Affiliations:** ^1^ Research Institute of Health Sciences (IUNICS) & Balearic Islands Health Research Institute Foundation (IdISBa), University of Balearic Islands (UIB), Palma, Spain; ^2^ Universidade Nove de Julho, São Paulo, Brazil; ^3^ Center for Mathematics, Computing and Cognition (CMCC), Federal University of ABC (UFABC), São Paulo, Brazil

**Keywords:** tDCS, fibromyalgia, transcranial electrical current stimulation, chronic pain, home-based tDCS

## Abstract

**Background:**

Fibromyalgia syndrome (FMS) is linked to central sensitization and neuroplastic alterations that contribute to chronic pain, fatigue, cognitive, sleep, and affective disturbances. Conventional treatments offer limited benefit. Non-invasive transcranial electrical stimulation (tES), particularly transcranial direct current stimulation (tDCS), may modulate brain function and relieve symptoms, but findings remain inconsistent.

**Objective:**

To systematically review and meta-analyze the effects of tES on clinical, neurophysiological, neuropsychological, and neurochemical outcomes in FMS.

**Methods:**

Seven databases were searched for studies published between April 2013 and April 2023. Eligible designs included randomized controlled trials, cross-over, one-arm, and case studies involving adult FMS patients. Data extraction followed Cochrane Collaboration guidelines and used RevMan 6.6.0.

**Results:**

Anodal tDCS produced short- to mid-term reductions in pain and mood symptoms, especially when applied over M1 or DLPFC. Longer interventions and repeated sessions enhanced effects, though protocol heterogeneity limited comparability. Both subjective (VAS, NRS) and objective (QST) measures confirmed pain reduction. Cognitive improvements were inconsistent, and quality of life effects were limited. Neurophysiological and neurochemical changes suggested possible mechanisms, though findings varied. Study quality was mixed, with small sample sizes and methodological inconsistencies. Meta-analysis revealed statistically significant but small effects on pain (Hedges' *g* < 0.2), with limited evidence on clinical relevance.

**Conclusions:**

Anodal tDCS may offer short-term relief of pain and mood symptoms in FMS, potentially through modulation of cortical excitability and neuroplasticity. However, due to variability in findings and methodological limitations, its clinical relevance remains unclear. Future trials should use standardized protocols, assess long-term effects, and include clinically meaningful outcome measures.

**Systematic Review Registration:**

https://www.crd.york.ac.uk/PROSPERO/view/CRD42023412332, PROSPERO CRD42023412332.

## Introduction

Fibromyalgia syndrome (FMS) is a complex and multifaceted chronic primary pain condition characterized by widespread musculoskeletal pain, chronic fatigue, cognitive impairment, a variety of somatic symptoms, and often co-occurring psychiatric conditions such as anxiety and depression (1–4). It predominantly affects women (80%–96%) and has a global prevalence estimated between 0.2% and 6.6% (5, 6). In addition to its high prevalence and multimorbidity, FMS is associated with significant social and economic burdens, including health inequities (7). A recent study reported that many healthcare providers lack adequate knowledge and tools to manage FMS, contributing to frustration among clinicians and negative healthcare experiences for patients (8, 9).

Management strategies for FMS include pharmacological and non-pharmacological treatments—such as physical exercise, cognitive behavioral therapy, lifestyle modifications, and complementary therapies (e.g., vitamin supplements, massage therapy) (10). However, the effectiveness of these interventions remains limited or inconsistent (11–22).

FMS is considered a central sensitization syndrome, as patients show abnormal sensory processing in both the central and peripheral nervous systems (4, 23, 24). They typically exhibit lower pain thresholds to both painful and non-painful stimuli compared to healthy individuals (25, 26). Neuroimaging and spectroscopy studies have identified structural brain changes and elevated glutamate (Glu) and glutamate plus glutamine (Glx) levels—markers associated with pain modulation, neurodegeneration, and apoptosis (27–31). A disruption in the balance between excitatory (glutamate/Glx) and inhibitory (GABA) neurotransmitters has been proposed as a key mechanism in FMS pathophysiology (28). These metabolic abnormalities are also linked to stress, posttraumatic stress disorder, and pain severity (32).

Transcranial electrical stimulation (tES) refers to a group of non-invasive neuromodulation techniques, the main types of which include transcranial direct current stimulation (tDCS), transcranial alternating current stimulation (tACS), and transcranial random noise stimulation (tRNS) (33, 34). These methods involve applying low-intensity electrical currents between electrodes on the scalp to modulate brain activity and promote adaptive neural responses (35, 36).

tDCS delivers a weak, constant, and unidirectional electrical current (1–2 mA) between a target electrode placed on the scalp and a reference electrode, typically positioned on an extracephalic site (anode or cathode, depending on the setup), to modulate neural excitability (37, 38). This stimulation induces polarity-specific changes in both spontaneous and evoked neuronal activity, where anodal polarization typically lowers the activation threshold through depolarization, increasing neuronal excitability, while cathodal polarization raises the threshold through hyperpolarization, leading to inhibition (39, 40). It is assumed that the brain structures located under the anode increase neural activity, whereas those underneath the cathode may reduce neural activity (40). However, the excitatory or inhibitory effects of tDCS are influenced by many factors beyond just anodal or cathodal polarity. These include the positioning of the electrodes, the orientation of neurons and axons in the brain, the degree of current conduction or impedance, the duration and intensity of stimulation, the initial neural activation state of the targeted areas, and individual anatomical variations such as skull thickness and gyral structure (39).

Unlike tDCS, which uses a constant current, tACS delivers an alternating current that oscillates at specific frequencies between electrodes (37, 41). Emerging research suggests that tACS can entrain and modulate brain activity, particularly in areas related to cognitive functions such as memory, attention, and executive control (42–44).

tRNS is a variation of tACS that applies an alternating current with a white noise frequency pattern (45, 46). Some evidence suggests that tRNS may enhance cortical excitability more effectively than both tDCS and tACS in healthy individuals (47, 48), but its effectiveness in chronic pain treatment remains unclear due to mixed findings (49, 50). Contributing to this ambiguity are variations in treatment protocols—including electrode placements, intensities, and session durations—and a lack of standardized guidelines, pointing to the need for rigorous research to clarify mechanisms and optimize clinical application.

In FMS, chronic pain is thought to arise from a loss of neural equilibrium caused by dynamic, plastic changes across widespread brain networks (35). Non-invasive tES - including tDCS, tACS, and tRNS - targets these disrupted processes by delivering low-intensity currents to specific brain regions such as the motor or prefrontal cortex.

Grounded in neurophysiological principles, this approach aims to modulate cortical excitability, improve neurochemical imbalances, and restore functional connectivity (51–53). Although exact mechanisms remain under investigation, noninvasive tES seeks to induce adaptive neuroplastic changes that alleviate symptoms like pain, fatigue, and cognitive dysfunction. It is important to note that non-invasive tES is a modulatory tool - not a cure - and works by enhancing the brain’s ability to reorganize maladaptive activity.

Despite promising findings and minimal side effects (55, 56), no systematic review has yet comprehensively assessed non-invasive tES in FMS by integrating outcomes across clinical, neurophysiological, and neurochemical domains. Existing reviews focus mostly on tDCS and its effects on pain and mood in randomized controlled trials (57–61), but methodological inconsistencies and high risk of bias limit their conclusions. This highlights the need for a comprehensive synthesis of the past decade of research to guide future clinical applications.

### Objective

The objective of this systematic review, supported by a supplementary meta-analysis, is to evaluate the extent of literature on the clinical, neurophysiological, and neurochemical effects of non-invasive tES in patients with FMS, with the following research questions: (1) Do adults with FMS who undergo non-invasive electrical current stimulation—anodal or cathodal tDCS, tACS, or tRNS—experience improvements in sleep problems, fatigue, quality of life, depression, anxiety, cognitive performance, or pain intensity? (2) What are the neurophysiological and neurochemical effects of anodal and cathodal tDCS, tACS, and tRNS in adults with FMS, as reflected by oscillatory activity, functional connectivity, and neurotransmitter levels? (3) What are the effect sizes of these significant clinical outcomes, and how might they inform the practical and clinical relevance of non-invasive tES interventions?

## Methods

To develop the research question and guide the literature search, the PICO framework was used (see Supplement 1, Appendix) (62). This systematic review follows the Cochrane Collaboration guidelines and reports results according to the PRISMA statement (63). Data extraction and editing were performed using Cochrane's Review Manager Software (RevMan, Version 6.6.0) (http://www.revman.cochrane.org; 64).

The methodological quality of included studies was assessed with the Physiotherapy Evidence Database (PEDro) scale, which provides a quick evaluation of clinical trial reliability and relevance for practice (65). A preliminary search in the Cochrane Database and PROSPERO found no existing or ongoing systematic reviews on this topic.

This review is registered on PROSPERO under the title: “Clinical and neurophysiological effects of noninvasive electrical brain stimulation in fibromyalgia”. More information is available at https://www.crd.york.ac.uk/prospero/display_record.php?ID=CRD42023412332.

### Objective and subjective pain measures

Pain assessment in FMS uses both objective and subjective methods. Objective measures evaluate physiological responses or sensory thresholds linked to pain perception, including electrocardiography, electroencephalography, neuroimaging, skin conductance, and Quantitative Sensory Testing (QST) (66). QST is a standardized tool commonly used in chronic musculoskeletal pain to measure sensory thresholds and pain responses (66–68). Subjective measures rely on patients' self-reports, with the Visual Analogue Scale (VAS) and Numeric Rating Scale (NRS) being the most common. The VAS ranges from 0 to 100, and the NRS from 0 to 10; both are equally effective for assessing chronic pain severity and disability, showing good agreement (69–71). While QST provides objective data on pain processing in FMS patients (25, 72, 73), the VAS and NRS capture the patient's personal pain experience (74, 75).

### Search strategy

The search strategy aimed to locate published studies, unpublished works, and gray literature. An initial limited search of PubMed was conducted to identify relevant articles. Keywords and index terms from these articles' titles and abstracts were then used to develop a comprehensive search strategy for databases including PubMed, Scopus, PsycINFO, Cochrane Controlled Trials Register, opengrey.eu, LILACS, and ClinicalTrials.gov. Accounts were created in each database to save searches for later retrieval. Search terms were entered only after the initial searches were completed. The search strategy was adapted for each database (see Supplement 2, Appendix for details).

References were exported to Mendeley, a reference management program, to efficiently identify and remove duplicates. After duplicates were excluded, the first screening pass was conducted by CW and HC, who reviewed study titles and excluded clearly irrelevant ones. This title-first screening was chosen to improve efficiency without sacrificing accuracy (76). Studies deemed potentially relevant by either reviewer moved on to the next stage.

The second pass involved screening abstracts of the selected titles. Finally, full-text reviews were conducted for articles still included after abstract screening. Reference lists of all included studies were also checked for additional relevant articles, which underwent the same three-step screening process (title, abstract, full text). This process continued until no further eligible studies were found.

Inter-rater agreement was calculated to assess consistency between reviewers. Only English-language studies published within the last decade (from April 1, 2013, to April 1, 2023) were included. For an overview of the search strategy, see Supplement 1, Appendix (PICO Worksheet and Search Strategy Protocol) (77–79).

### Inclusion criteria

Primary quantitative research studies were included if they met the following criteria:

(1) Peer-reviewed original studies on noninvasive tES in FMS (e.g., longitudinal studies, pilot studies, pilot randomized controlled trials, randomized controlled trials, clinical quasi-experimental trials, single-case or small group designs, and uncontrolled or controlled pre-posttest studies);
(2) Adult participants (≥18 years) with a medical diagnosis of FMS (80);
(3) Published in English.

### Exclusion criteria

Studies were excluded if they met any of the following criteria:

(1) Review articles or meta-analyses;
(2) Comments, editorials, letters, or meeting/congress abstracts;
(3) Non-English publications;
(4) Samples with secondary musculoskeletal pain, no formal diagnosis of FMS, or any study not meeting the inclusion criteria.

### Source of evidence selection

General search terms used included: transcranial direct current stimulation, fibromyalgia, treatment, sleep, cognitive functioning, pain intensity, depression, anxiety, quality of life, EEG, oscillatory activity, and functional connectivity. The searches were conducted on April 14th and 15th, 2023, yielding a total of 1,203 articles: 241 from PubMed, 58 from LILACS, 120 from Scopus, 406 from the Cochrane Controlled Trials Register, 248 from ClinicalTrials.gov, and 130 from PsycINFO.

All identified citations were collected and uploaded into Mendeley, where 808 duplicates were removed. The remaining 395 articles were screened against the review's inclusion criteria, resulting in 312 exclusions. Of the 83 articles left, abstracts were further screened, and 38 were excluded. Forty-five articles were retrieved in full as potentially relevant.

The full texts of these 45 articles were assessed in detail against the inclusion criteria. Reference lists of the 30 articles that met criteria were also screened, and 2 additional studies were included. All full-text citations were collated and uploaded to Mendeley.

**Figure 1:**
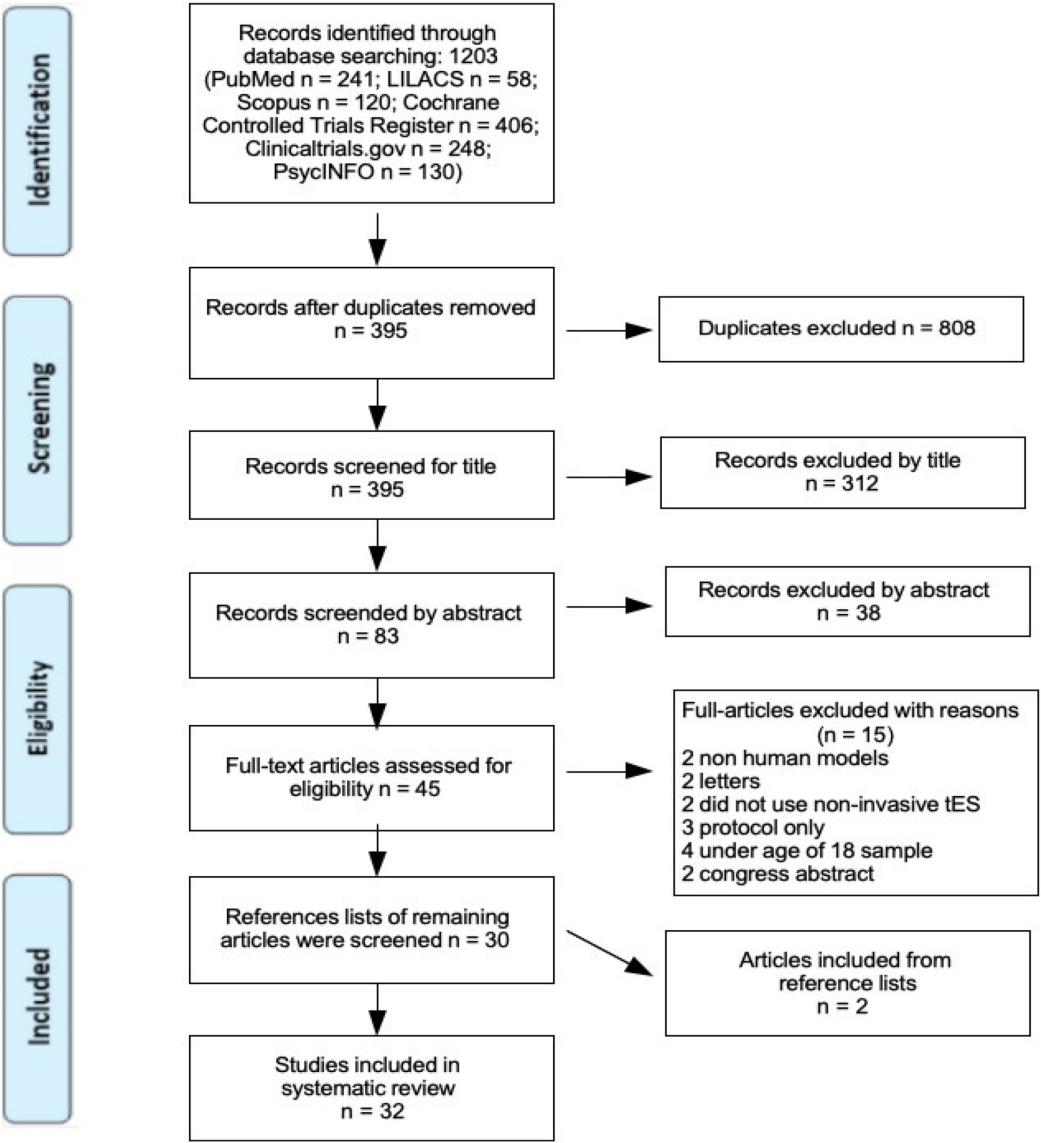
PRISMA flow diagram, search and study inclusion process. Page et al. (63).

The search results and study inclusion process are presented in a PRISMA flow diagram (Figure 1) (63).

### Validity assessment

Besides the primary quantitative studies mentioned earlier, no additional inclusion criteria were applied. The quality of each study was assessed using different tools depending on the study design. For randomized controlled trials (RCTs), the Cochrane Risk of Bias tool (81) and the PEDro scale were used (82). Within-subject crossover and single-arm studies were evaluated with the Cochrane Collaboration's risk-of-bias tool adapted for such designs (MINORS RoB; see Supplement 6, Appendix). For the case study, a single-case design risk of bias tool developed by Reichow, Barton, and Maggin (83) was applied (84).

Risk of bias assessments addressed both internal and external validity. Two authors (CW and HC) independently evaluated each study using the appropriate tool based on study design. Inter-rater reliability was calculated to assess consistency, and any discrepancies were resolved through discussion or, if needed, in consultation with a third author (PM). Inter-rater reliability was high, with a Cohen's kappa of 0.96 (*κ* = 0.9588), indicating excellent agreement between reviewers.

### Data extraction

For each study, one author (CW) selected validated psychometric data collected before and after intervention, including measures of pain intensity, quality of life, depression, pain pressure threshold, heat pain threshold, and heat pain tolerance. Numerical data were then extracted for change analyses.

Data extraction was performed using the Cochrane review software RevMan (version 6.6.0) alongside the PEDro scale developed by the Institute for Musculoskeletal Health at the University of Sydney, Australia (https://www.pedro.org.au) (see Supplement 4, Appendix). Both tools were applied to assess different types of bias, such as selection bias, attrition bias, and blinding. For randomized controlled trials, intention-to-treat analyses were examined to evaluate how investigators handled participant dropouts (85, 86).

The extracted data included details about study design, sample size, treatment and control group characteristics, and outcomes relevant to the review questions. To balance the exploratory nature of this systematic review with evidence quality, quality assessment was conducted using RevMan.

Bias assessment of included crossover studies followed the method quality review by Ding et al. (87), based on the Cochrane Handbook and expert commentary (see Supplement 5, Appendix).

## Quantitative data synthesis

For effect size measurements and outcomes see Supplement 7, Appendix.

## Results

The inter-rater reliability was calculated and resulted in Cohen's Kappa of 0.96, which is an indication for an almost perfect agreement (88) (see Supplement 3, Appendix, for more detail). For a detailed summary of the treatment characteristics and study designs of the included studies, please refer to Supplement 3, Appendix.

### Key study characteristics and treatment protocols

This review includes 32 studies with a total of 1,351 participants, of whom 98% were female. Sample sizes varied widely, from a single-case report to studies with up to 130 participants. Regarding study design, most were randomized controlled trials (*n* = 23; 71%), followed by within-subject crossover designs (*n* = 6; 18.75%). There were also two non-randomized clinical trials and one single-case study.

Most interventions (90.6%) took place in laboratory settings, while only three studies (9.4%) used home-based tDCS protocols. Anodal tDCS was by far the most common stimulation type (*n* = 31), with only two studies testing cathodal tDCS. One study explored anodal tRNS, and no studies used tACS in fibromyalgia patients.

The primary motor cortex (M1) was the most frequently targeted brain region (*n* = 21), followed by the dorsolateral prefrontal cortex (DLPFC) (*n* = 13). Some studies also stimulated the operculo-insular cortex or applied occipital nerve stimulation.

Most studies used a stimulation intensity of 2 mA (*n* = 27), typically for 20 min per session (range: 10–30 min). The number of sessions varied widely, from 1 to 60, though most protocols involved between 1 and 5 sessions. Sham control conditions were included in 87% of studies, often involving short-duration stimulation (5–30 s) with the same electrode setup as active conditions.

Nine studies combined tDCS with other interventions such as aerobic exercise, low-dose naltrexone, or repetitive transcranial magnetic stimulation, providing insight into potential synergistic effects (e.g., 89).

### Effects of intervention—synthesis of clinical symptoms outcomes

Among the 33 studies assessing clinical symptom improvement in FMS, all but one applied anodal tDCS with current intensities between 1 and 2 mA. A clear trend toward pain reduction emerged, with 89% of studies reporting significant improvements in pain-related measures following non-invasive tES.

Stimulation over the left M1 or DLPFC increased pain thresholds and tolerance, with both anodal and cathodal stimulation showing efficacy. This matches findings from Teixeira et al. (60), who found no significant difference in pain outcomes between M1 and DLPFC stimulation sites. The diffuse stimulation effect of tDCS may explain this, as it likely modulates both descending pain inhibitory circuits and affective-cognitive pathways simultaneously.

This non-focal mechanism has important clinical implications. If tDCS does not require highly precise targeting, clinicians can tailor stimulation based on individual symptoms—for example, M1 stimulation for sensory symptoms and DLPFC for cognitive-affective symptoms. However, overlapping effects complicate the understanding of specific neural mechanisms. Future studies should consider more focal methods—such as high-definition tDCS or neuronavigation-guided repetitive transcranial magnetic stimulation—and combine these with neuroimaging to clarify mechanisms (90, 91). The diffuse nature of standard bipolar tDCS may also contribute to inconsistent trial outcomes, as individual neuroanatomy and symptom profiles likely affect responses.

Interestingly, only anodal tDCS was linked to increased mechanical detection thresholds, indicating enhanced tactile sensitivity. This suggests that different current polarities and montages produce distinct sensory effects, highlighting the need to tailor protocols to symptom dimensions and goals.

Though anodal tDCS shows promise, its effects are often short- to medium-term. Several studies reported meaningful pain and mood improvements, especially with repeated sessions (92–95). For example, Brietzke et al. (92) and Khedr et al. (93) observed up to a 46% pain reduction and significant mood improvements following repeated M1 stimulation. However, benefits typically diminish after treatment ends, demonstrating the challenge of sustaining effects over time.

Teixeira et al. (60) provide critical insight here: their meta-analysis indicates that not just the number of sessions, but their distribution over time, influences outcomes. Protocols lasting 4 weeks or longer yielded greater analgesic effects—even with similar total session numbers—suggesting a cumulative neuroplastic mechanism. Supporting this, Monte-Silva et al. (96) showed that M1-tDCS with inter-session intervals exceeding 24 h produced more robust cortical excitability changes. In fibromyalgia, the most clinically relevant improvements appeared after 4 weeks of treatment (60), paralleling findings in depression and stroke rehabilitation where longer stimulation yields stronger outcomes (97).

Consistent with this, Brietzke et al. (92) reported nearly 50% pain reduction after 20 sessions, increasing to 60% after 60 sessions, which reinforces the idea that effects accumulate with repeated stimulation and supporting longer-term interventions for sustained relief.

Combining tDCS with other treatments has shown mixed results. Mendonca et al. (98) found that pairing tDCS with aerobic exercise improved pain, mood, and anxiety, but other studies reported no added benefit. For example, Matias et al. (99) found no advantage when tDCS was combined with functional exercise, and similar null effects occurred with mindfulness or occipital nerve stimulation (100, 101). Variability may result from differences in timing, intensity, or individual responsiveness, suggesting not all combination approaches are equally effective.

Comparing stimulation sites, M1, DLPFC, and operculo-insular cortex stimulation led to short-term improvements in pain and fatigue (102), but benefits were not sustained. In contrast, high-definition tDCS protocols (e.g., 103) produced rapid pain relief, indicating that more focal stimulation may enhance short-term outcomes. These findings highlight the importance of both stimulation site and protocol design for clinical effectiveness.

tDCS has also shown promise for cognitive and affective symptoms. Over half of the studies addressing mood reported reductions in depression (e.g., 93, 104). Several trials noted cognitive improvements—especially in attention, memory, and executive function—when tDCS was combined with cognitive training (105). These effects may reflect enhanced neuroplasticity from activating cognitive circuits during stimulation.

However, improvements in quality of life were less consistent. Some studies suggested placebo effects or no change (e.g., 106), possibly due to varying definitions of quality of life or differences in disease progression among participants.

Overall, anodal tDCS shows potential for relieving pain, mood symptoms and fatigue (95) in fibromyalgia, but its long-term effectiveness remains uncertain. Treatment outcomes depend on protocol duration, session spacing, stimulation site, and patient factors. For clinical use, flexible yet structured protocols—ideally lasting several weeks—may offer the best results. Emerging technologies like high-definition tDCS, closed-loop systems, and personalized montages could improve treatment precision and durability.

Future research should explore optimal combinations with behavioral therapies and examine broader outcomes such as cognition and quality of life, to better tailor and enhance treatments for FMS.

### Effects of intervention—synthesis of neurophysiological and neurochemical outcomes

Out of the 32 studies included in this review, 13 examined neurophysiological and/or neurochemical outcomes of non-invasive brain stimulation in patients with FMS, mainly using anodal tDCS, and in one case, tRNS. These studies investigated a wide range of biomarkers and brain activity patterns, including brain derived neurotrophic factor (BDNF), Glx, GABA, β-endorphins, motor and somatosensory evoked potentials, cortical oscillations (alpha 2), blood-oxigen-level-dependent (BOLD) variability, resting-state functional connectivity, and hemodynamic activity.

### Neurochemical markers: BDNF, β-endorphins, and brain metabolites

A key focus was on BDNF as a marker of neuroplasticity and its modulation by tDCS. The findings were somewhat mixed. Brietzke et al. (92) and Caumo et al. (107) reported that higher baseline BDNF levels were associated with greater pain relief following tDCS. In contrast, Santos et al. (108) and Serrano et al. (105) observed opposite effects, where elevated baseline BDNF correlated with reduced cognitive and analgesic improvements. Despite these contradictions, the studies collectively suggest that BDNF levels—whether measured at baseline or as a dynamic response to treatment—may influence individual responsiveness to tDCS, likely through effects on synaptic plasticity.

Similarly, Khedr et al. (93) found that the analgesic and mood benefits of M1 stimulation were linked to increased serum β-endorphin levels. These β-endorphin increases were negatively correlated with symptom severity and positively correlated with pain threshold, supporting the involvement of endogenous opioid release in tDCS-induced pain modulation.

From a neurochemical perspective, Foerster et al. (109) used magnetic resonance spectroscopy and found that anodal tDCS over M1 significantly reduced Glx concentrations in the anterior cingulate cortex, a critical area for pain processing. Notably, baseline Glx levels predicted treatment response, underscoring the importance of pre-existing neurochemical states in determining tDCS outcomes.

### Functional connectivity and cortical dynamics

Several studies examined how tDCS affects resting-state functional connectivity and cortical oscillations. Cummiford et al. (110) found that anodal tDCS over M1 altered functional connectivity within thalamic and sensorimotor networks. They also showed that stronger baseline connectivity between M1 and pain-related brain regions predicted greater pain relief. These changes might reflect disruption of thalamocortical signaling, potentially mediated by endogenous opioid systems. Similarly, Lim et al. (94) used BOLD signal variability as an indicator of dynamic brain function. They showed that anodal tDCS increased neural variability in prefrontal and cingulate areas, while reducing it in pain-related regions such as the posterior insula. These neural changes were linked to pain relief, suggesting that signal variability could predict how well patients respond to treatment. De Melo et al. (111) focused on cortical oscillations and found that tDCS reduced alpha-2 frequency activity in frontal and parietal regions, an abnormality commonly seen in FMS. Although this did not differentiate pain outcomes between sham and active stimulation groups, only anodal tDCS influenced alpha oscillations, indicating specific neuromodulatory effects.

### Evoked potentials and cortical excitability

Mendonca et al. (98), Schein et al. (112), and Terrasa et al.^1^ studied cortical excitability through evoked potentials. Mendonca et al. (98) observed no significant changes in motor evoked potentials (MEP), inter-cortical inhibition, or inter-cortical facilitation after tDCS alone or combined with aerobic exercise. However, they did note improvements in mood and pain, suggesting these effects may be mediated through non-motor pathways. Schein et al. (112) compared tDCS with Hypnotic Analgesia Suggestion. They found that while Hypnotic Analgesia increased pain tolerance, anodal tDCS over the DLPFC increased MEP amplitudes and reduced short-interval inter-cortical inhibition. This suggests enhanced corticospinal excitability and indicates that these two interventions might work through different but complementary pain regulation pathways. Terrasa et al. (see text footnote 1) investigated somatosensory gating and showed that a single session of anodal tDCS over the primary somatosensory cortex (S1) modulated late somatosensory evoked potentials differently across hemispheres. Specifically, it enhanced inhibition in the right hemisphere and reduced it in the left. Although the significance of this lateralization is unclear, the findings demonstrate that tDCS can influence higher-order cognitive processing of somatosensory information.

### Hemodynamics and cortical metabolism

Using functional near-infrared spectroscopy, Rocca et al. (113) demonstrated that one session of M1 tDCS counteracted cortical hypometabolism during motor tasks in FMS patients, particularly by increasing oxygenated hemoglobin in motor regions. These effects were specific to FMS patients and absent in healthy controls, suggesting that tDCS may help normalize dysfunctional metabolic activity in motor-related cortical networks.

### Cognitive and attention networks

Beyond pain modulation, Santos et al. (108), Serrano et al. (105), and Silva et al. (114) investigated the cognitive effects of DLPFC stimulation. Their results consistently showed improvements in working memory, attention, and executive functions, especially when tDCS was paired with cognitive tasks. These gains were linked to baseline neuroplasticity markers, such as BDNF, suggesting a synergy between neuromodulation and task-based plasticity enhancement.

### Combined and adjunctive interventions

Paula et al. (115) examined a combined treatment using low-dose naltrexone (LDN) and tDCS. Surprisingly, analgesic effects appeared across multiple groups, including placebo, highlighting strong placebo responses and the complexity of disentangling specific mechanisms in combined interventions. The interaction between opioid modulation via LDN and tDCS-induced plasticity remains unclear. Together, these studies offer preliminary but compelling evidence that tDCS induces neurochemical and neurophysiological changes in FMS patients. Key findings suggest BDNF and Glx levels may act as state markers predicting treatment responsiveness; resting-state functional connectivity and neural signal variability reflect brain-wide changes linked to analgesia; tDCS modifies cortical excitability, hemodynamics, and somatosensory gating, though outcomes depend on stimulation site, duration, and protocol. Cognitive and affective improvements may arise from stimulation-induced plasticity, particularly when combined with training. Despite outcome variability and some inconsistencies, a consistent theme is the importance of baseline brain state—neurochemistry, connectivity, or signal variability—as a predictor of tDCS benefit. Future research using multimodal imaging, larger samples, and personalized protocols (e.g., MRI-guided tDCS) may better elucidate and leverage these mechanisms clinically.

### Effects of intervention—neuropsychological outcomes

Neuropsychological outcomes, particularly cognitive function, were assessed in seven studies, with five reporting significant improvements following neuromodulation (49, 92, 105, 107, 114). These findings suggest potential cognitive benefits of non-invasive brain stimulation in FMS, especially in domains affected by “fibrofog,” such as memory, attention, and executive function. Curatolo et al. (49) evaluated high-frequency tRNS, applied over the left M1. Their intervention consisted of 10 sessions (1.5 mA, 101–640 Hz, 10 min) across 2 weeks. Alongside improvements in pain and mood, participants receiving active tRNS showed enhanced cognitive performance, particularly in attention, verbal learning, and executive functioning—areas frequently impaired in FMS. Similarly, Santos et al. (108) demonstrated that anodal tDCS over the DLPFC combined with working memory training was more effective than cognitive training alone in improving immediate memory and verbal fluency in female FMS patients. Notably, the cognitive effects of tDCS were partly moderated by baseline BDNF levels, indicating that individual neuroplasticity profiles influence treatment responsiveness. In the active group, higher baseline BDNF correlated with greater improvements in the Rey Auditory Verbal Learning Test, while in the placebo group, BDNF was only associated with changes in short-term digit span memory, suggesting different underlying mechanisms for tDCS and training alone (Tables 1, 2).

**Table 1:** Characteristics of selected studies.

	Author	Year	*n*	Design	Objective	Types of intervention		Clinical variables	Neuro-physiologial variables	Quantitative sensory assessment	Setting	Follow-up	Primary outcomes
						tDCS	Other						
1	Arroyo-Fernández et al. (89)	2022	120 (113 ♀)	Randomized, triple-blind, sham-controlled trial, between subjects design	To evaluate effectiveness of tDCS combined with physical exercise in FMS.	Anodal tDCS over left M1 (C3); 2 mA; 20 min/sham tDCS; total of 5 sessions in 2 weeks	Aerobic & muscle strengthening exercise	VAS, FIQ, STAI, PCS, BDI-II	-	PPT	Laboratoy	1 month	Active and sham tDCS improved health status, pain catastrophizing, and depression vs. control, but pain intensity only decreased in active tDCS.
2	Brietzke et al. (92)	2020	20 ♀	Randomized, double-blind controlled trial, between subjects design	To examine if 60 Home-based anodal tDCS over DLPFC is more effective than sham tDCS to improve widespread pain and pain related disability.	Anodal & sham tDCS over left DLPFC; 2 mA; 30 min; 60 sessions for 5 consecutive days/12 weeks	-	VAS, FIQ, STAI-E-T, CSI-BP, BDI-II, BP-PCS, PSQI, B-PCP:S, BDNF serum levels	-	HPT, PPT,	Home-based	-	Sixty sessions of anodal tDCS treatment for FMS induced large pain decreases. Home-based tDCS is a feasibile method of intervention for FMS. Brain-derived neurotrophic factor to index neuroplasticity
3	Castillo-Saavedra et al. (116)	2016	20 (17 ♀)	Open-label, single arm study	To define a methodology for a clinical effective treatment of pain in FMS.	Anodal HD-tDCS over C3; 2 mA; 20 min; 10 sessions for 5 consecutive days/2 weeks	-	VAS, pain diary, FIQ, BDI-II	Heat stimulation, EEG recording, BNA analysis	Semmes- Weinstein Monofilaments, PPT	Laboratory	2 and 8-week	Fifty percent pain reduction in 50% of the sample, and improvement QoL with a 6-week response rate of 50%. The median number of HD-tDCS to reach clinically meaningful results is 15 sessions.
4	Caumo et al. (107)	2022	48 ♀	Randomized, double-blind, sham controlled trial, between subjects design	To compare the effect of Home-based anodal tDCS, with sham-tDCS to reduce scores on Pain Catastrophizing Scale (PCS) and disability-related to pain (DRP).	Anodal & sham tDCS bifrontal over left DLPFC; 2 mA, 20 min; 20 sessions; 5 consecutive working days × 4 weeks	-	PCS, PCP:S, BDI-II, PSQI, FIQ, STAI, PNS, CSI	-	HPT, HPTo, BDNF serum levels	Home-based	-	Home-based bifrontal a-tDCS over the left DLPFC were linked to (i) Decreased rumination and magnification of pain catastrophizing, (ii) Improved the disability for daily activities due to FMS symptoms. The findings support the feasibility of self-applied Home-based tDCS on DLPFC to improve FMS symptoms.
5	Cummiford et al. (110)	2016	13 ♀	Within-subjects cross-over design	To assess how tDCS alters resting state functional connectivity & how changes relate to clinical pain.	Anodal & sham tDCS over C3; 2 mA; 20 min, 5 sessions for 5 consecutive days	-	VAS	resting state fMRI/seed to whole brain FC	-	Laboratory	-	Repetitive anodal tDCS over C3 resulted in changes in FC that last beyond the stimulation period and may produce analgesia by altering thalamic connectivity.
6	Curatolo et al. (49)	2017	20 ♀	Randomized, sham controlled study, between subjects design	To investigate clinical effects of M1 tRNS stimulation in FMS.	High frequency (HF) & sham tRNS over C3; 1.5 mA in 101–640 Hz range; 10 min; for 5 consecutive days during 2 weeks	-	VAS, FIQ, HADS, TMT, RAVLT, FBDST, FAS	-	-	Laboratory	-	Anodal tRNS over M1 resulted in general improvement of FMS symptoms (pain, depression, anxiety, FIQ, TMT (A), RAVLT & FAS scores.
7	DallAgnol et al. (84)	2015	1 ♀	Case study	To examine clinical effects of tDCS in FMS.	Anodal tDCS over M1 & DLPFC & sham; 2 mA; 20 min; 10 sessions for 5 consecutive working days	-	VAS, FIQ-B, STAI, BDI-II, PCS-B	-	-	Laboratory	-	Decrease in pain perception and anxiety levels, as well as ruminative catastrophism.
8	de Melo et al. (111)	2020	31 ♀	Longitudinal, randomized, double-blind, sham controlled study, between subjects design	To compare the effect of 2 anodal tDCS protocols on pain levels.	Anodal & sham tDCS; 2 mA, 20 min over left M1 during 5 consecutive working days	-	CIRS, VAS, MMSE, BDI, BAI	-	-	Laboratory	1 week	Reduction in pain intensity after treatment for 5 and 10 consecutive days, resprectively.
9	Fagerlund et al. (117)	2015	48 (45 ♀)	Randomized controlled trial, between subjects design	To investigate clinical effects of anodal tDCS in FMS.	Anodal & sham tDCS; 2 mA; 20 min; over M1; on 5 consecutive days	-	FIQ, HADS, SCL-90R, SF36	-	-	Laboratoy	1 month	Improvement in pain and FM-related daily functioning.
10	Foerster et al. (109)	2015	12 ♀	Within subjects cross-over longitudinal trial, within subjects design	To explore neurochemical action in brains of FMS patients using proton magnetic resonance spectroscopy.	Anodal & sham tDCS over left M1 for 2 × 5 consecutive days with a wash-out period of 1 week after first 5 days; 2 mA; 20 min	-	VAS, PANAS, McGill	-	-	Laboratoy	-	GABA, Glx and NAA play important role in pathophysiology of FMS and affects modulation by tDCS. Baseline Glx levels in anterior cingulate predicted response to treatment.
11	Forogh et al. (104)	2021	30 ♀	Randomized controlled study, between subjects design	To compare the effects of rTMS and tDCS on pain & QoL.	Anodal tDCS; 3 × 2 mA, 20 min over left DLPFC; total of 3 sessions during 1 week	10 Hz rTMS	VAS, DASS-21, FIQR	-	-	Laboratory	6 and 12 weeks	rTMS as well as tDCS resulted in significant changes in pain intensity and anxiety/depression. However, three sessions of rTMS over DLPFC had a significantly greater & longer lasting analgesic effect compared to tDCS.
12	Jales et al. (118)	2015	20 ♀	Prospective, double-blind, randomized controlled study with parallel arms, between subjects design	To evaluate effect of tDCS on pain and QoL in FMS.	Anodal & sham tDCS; 1 mA; 20 min; over left M1; 1 × week over 10 consecutive weeks	-	VAS, SF-36, FIQ	Single Photon Emission Computer Tomography (SPECT)	Tender point pain measurement by Fischer Algiometer	Laboratory	-	tDCS proved to be effective in clinical pain control and improvement in QoL.
13	Kang et al. (55)	2020	46 (44 ♀)	Not randomized clinical single arm trial	To examine the efficacy and safety of tDCS as an add-on treatment in FMS.	Anodal tDCS; 2 mA; 20 min; over left M1 during 5 consecutive days	-	VAS, FIQ, BPI, BFI, BDI, STAI-I, STAI-II, MOSS-SS	-	-	Laboratory	4 weeks	Significant relief in pain which may be an effective add on treatment. tDCS may play an important role in improving chronic pain, depression, and fatigue in FM patients.
14	Khedr et al. (93)	2017	40 (34 ♀)	Randomized double-blind clinical trial, between subjects design	To evaluate the effects of tDCS in relieving FMS pain and its relation with beta-endorphin changes.	Anodal & sham tDCS, 2 mA, 20 min over left M1 for 10 consecutive working days	-	VAS, WPI, SS, HAM-D, HAM-A	-	Mechanical pain sensitivity threshold with Frey technique	Laboratory	2 weeks and 1 month	Ten sessions of anodal tDCS over M1 induced pain relief and mood improvement in patients with FMS which were found to be related to changes in serum endorphin levels.
15	Lim et al. (94)	2021	12 ♀	Longitudinal trial within-subjects cross-over design	To examine if M1-tDCS modulates regional temporal BOLD signals and if this is associated with pain improvement.	Anodal & sham tDCS over left M1; 2 mA; 20 min; sham tDCS during 5 consecutive days, 7–11 days of wash-out period, then anodal tDCS	-	VAS, McGill	BOLD, fMRI	-	Laboratory	-	tDCS over left M1 might reverted temporal variability of fMRI signals in the rACC/vmPFC and posterior insula linked to FMS pain.
16	Loreti et al. (95)	2023	35 ♀	Randomized triple-blind clinical trial, between subjects design	To examine analgesic effects of anodal tDCS over M1 in FMS.	Anodal & sham tDCS over left M1; 2 mA; 2 × 13 min with a 20 min break in between for 10 sessions (2 × 5 consecutive days)	-	VAS, SOPA-B, FIQ, WHOQOL, HAM-A, HAM-D, FAS	-	-	Laboratory	30 days and 90 days	Pain and fatigue decreased and QoL improved in patients with FMS.
17	Matias et al. (119)	2022	30 ♀	Double-blind parallell, randomized, sham controlled trial, between subject design	To assess the pain-related difference between right-left side of the body after 5 sessions of tDCS in women with FMS.	Anodal & sham tDCS anodal over left M1, 2 mA; 20 min, 5 consecutive days	-	VAS, FIQ, NRS, HAS	-	-	Laboratory	-	Five sessions of anodal tDCS over the left M1 improved pain in women with FMS. No differences between right-left body sides were found.
18	Matias et al. (99)	2022	47 ♀	Randomized, double-blind, sham-controlled trial, between subjects design	To examine the effects of tDCS and functional exercise on pain, functional performance, psychological symptoms and QoL in FMS patients.	Anodal & sham tDCS; 2 mA, 20 min over left M1; 5 × during first week followed by FE on 3 of 5 days	Functional exercise 3 × week × 8 weeks	VAS, 6MWT, QoL, BDI, MFI, Affective valence by the Feeling Scale	-	PPT	Laboratory	-	Pain intensity decreased, functional performance, psychological symptoms, and quality of life increased significantly in both groups. No significant differences between groups were found in all outcomes. tDCS combined with functional exercises did not enhance the effects of physical exercise on pain, functional performance, psychological symptoms, & QoL in FM patients.
19	Mendonca et al. (98)	2016	45 ♀	Sham-controlled randomized clinical trial, between subjects design	To examine if intervention of tDCS and aerobic exercise induces significantly greater pain reduction as compared to tDCS alone and aerobic exercise alone.	Anodal & sham tDCS over left M1; 2 mA, 20 min, 5 consecutive days	Aerobic exercise 30 min 3 days peer week	VNS for anxiety, SF-36, BDI-II	-	PPT	Laboratory	2 months	The combination intervention of tDCS and AE had a significant effect on pain, anxiety, and mood.
20	Paula et al. (115)	2022	86 ♀	Randomized, double-blinded, parallel, placebo/sham-controlled trial, between subjects design	To evaluate the analgesic and neuromodulatory effects of a combination of low-dose naltrexone (LDN) and tDCS in patients with FMS.	LDN & anodal tDCS/LDN & sham tDCS/placebo & anodal tDCS/placebo & sham tDCS; tDCS over M1 contralateral to the dominant cortex; 2 mA, 20 min; total of 5 sessions on 5 consecutive days	Low-dose naltrexone (LDN)	VAS, FIQ, BDI-II, STAI, PCS, PCP: S	-	PPT	Laboratory	-	Combined LDN + tDCS has possible benefits in reducing pain frequency and intensity;however, a placebo effect was observed in pain using VAS, and further studies should be per-formed to analyze the possible association
21	Ramasawmy et al. (100)	2022	30 (28 ♀)	Double-blinded, randomized and sham-controlled pilot clinical trial, between subjects design	To address the efficacy of mindfulness meditation and tDCS for pain and associated symptoms in patients with FMS.	Anodal & sham tDCS over left M1, 2 mA, 20 min, 10 sessions during 2 × 5 consecutive days	Body scan exercise followed by 20 min of guided mindfulness meditation	NRS, FIQ, DASS, NRS, FMI	-	PPT	Laboratory	4 weeks	Combined anodal tDCS and mindfulness meditation did not improve clinical pain and associated symptoms in FMS patients.
22	Rocca et al. (113)	2022	76 ♀	Randomized, sham controlled trial, between subjects design	To examine if one tDCS session can restore the reduced metabolism in FMS patients.	Anodal & sham tDCS over left M1 (C3), 2 mA, 20 min; single session	-	WPI, FIQ, SS	fNIRS to measure hemodynamic variation (oxyhemoglobin levels), movement speed (finger tapping)	-	Laboratory	-	A single session of anodal tDCS of the corticalmotor network was able to restore basic cortical hypometabolism in FMS.
23	Samartin-Veiga et al. (106)	2022	130 ♀	Randomized, sham-controlled, double-blind clinical trial, between subjects design	To establish the optimal area of stimulation, comparing the 2 classical targets (M1 & DLPFC) and a novel pain-related area, the operculo-insular cortex.	Anodal & sham tDCS sessions, 2 mA, 20 min, left M1, DLPFC, OIC; 15 sessions on 5 consecutive working days during 3 consecutive weeks	-	FIQ, HADS, MFE, PSQI, NRS	-	PPT	Laboratory	6 months	Active tDCS is not superior to sham stimulation in improving QoL in FMS
24	Samartin-Veiga et al. (102)	2022	130 ♀	Double-blind, placebo-controlled study, between subjects design	To examine how active or sham tDCS over M1, DLPFC, & OIC affect QoL in patients with FMS.	Anodal & sham tDCS sessions, 2 mA, 20 min, left M1, DLPFC, OIC; 15 sessions on 5 consecutive working days during 3 consecutive weeks	-	FSQ, FIQ-R, SF-36	-	-	Laboratory	6 months	Irrespective of cortical target and anodal or sham tDCS, there was significant improvement in QoL, immediately after the end of treatment and 6 months later. Placebo effect might be explained by positive expectations on efficacy of neuromodulation techniques and non-specific psychosocial variables.
25	Santos et al. (108)	2018	40 ♀	Randomized, double-blind, sham-controlled, clinical trial, between subjects design	To test the effects of eight sessions of tDCS and cognitive training on immediate and delayed memory, verbal fluency, working memory and its association with BDNF levels.	Anodal tDCS & sham, 2 mA, 20 min over left DLPFC for 8 consecutive working days	WMT	VAS, COWAT, WMT, RAVLTS, PASAT, FDS, BDS, BDI-II, STAI-II, BP-PCS, B-PCP:S, PSQI, MINI-DSM-IV	CSI, BDNF	HPTh, HPTo	Laboratory	-	When compared to cognitive training alone or sham, the combination of WMT & active tDCS increased immediate memory capacity and verbal fluency. The effect of active tDCS on short-term memory partially depended on baseline levels of serum BDNF.
26	Schein et al. (112)	2023	18 ♀	Cross-over randomized clinical trial, within subjects design	To compare the effect of HAS, anodal tDCS on the left DLPFC, and CPT, HPTh, MEP, SICI, ICF, and CSP.	Anodal tDCS over the l-DLPFC, 2 mA, 20 min; single session	Hypnosis	BDI-II, STAI, PSQI, BP-PCS, CSI	RMT, MEP, CSP, SICI, ICF	-	Laboratory	-	HAS compared to anodal tDCS increased the pain tolerance with a moderate effect size (ES). While compared to rest-testing, HAS increased the CPT with a large ES. The anodal tDCS compared to HAS increased the MEP amplitude with large ES. Likewise, it's ES compared to rest-testing in the MEP size was large. These findings revealed that HAS affects contra-regulating mechanisms involved in perception and pain tolerance, while the a-tDCS increased the excitability of the corticospinal pathways.
27	Serrano et al. (105)	2022	36 ♀	Randomized, double-blind, parallel, sham-controlled clinical trial, between subjects design	To evaluate the efficacy and safety of home-based tDCS on cognitive impairment in FMS.	Anodal & sham tDCS over left DLPFC (F3), 20 sessions during 4 weeks (5 consecutive days), 2 mA, 20 min	-	FIQ, PCS, BDI-II, PSQI, Pain NPS, WM, TMT A-B, COWAT, Digits Subtest from WAIS-III,	BDNF	PPT	Home-based	-	Daily treatment with anodal tDCS over the left DLPFC for 4 weeks improved cognitive impairment in FMS. The effect of anodal tDCS was related to the severity of dysfunction of the Descendant Pain Modulation System and changes in neuroplasticity state. Concretely, executive and divided attention, working memory, and cognitive flexibility, as well as verbal semantic and phonemic fluency and QoL improved. These changes were positively related to the average percentage changes of the BDNF as compared to baseline. The decrease in BDNF linked to anodal tDCS was associated with an improvement in FMS core symptoms (depression, pain catastrophizing, sleep quality).
28	Silva et al. (114)	2017	40 ♀	Randomized, crossover blind, clinical trial, within subject design	To test the effects of a single session anodal tDCS coupled with a Go/No-go task on alertness, orienting, executive control, and pain in FMS.	Anodal & sham tDCS, 1 mA, 20 min over DLPFC, single session	-	BDI-II, VAS, STAI, SCID-I, PSQ, PCS,	-	HPTh, HPTo	Laboratory	-	One session of anodal tDCS over the left DLPFC has a modulatory effect on the orienting and executive attentional networks. The effect on executive attention has been shown to be independent of the effect on pain.
29	Terrasa et al. (see text footnote 1)	2025	39 ♀	Randomized, double-blind, sham controlled trial, between subjects design	To examine the modulatory effects of anodal tDCS applied over the left S1 on sensory gating during repetitive touch stimulation in FMS patients.	Anodal & sham tDCS over S1 (CP3), 1.5 mA, 20 min, single session	-	BDI, STAI, TAS-20, FIQ, McGill Pain Inventory, POMS, VAS-tDCS, WHYMPI	-	PPT	Laboratory	-	A single short session of anodal tDCS modulation led to enhancement of sensory gating contralateral to the stimulated hemisphere, while worsening sensory gating ipsilateral to the stimulation side related to the late stages of the sensory process. Anodal tDCS failed to modulate sensory gating in the early stages of information processing.
30	To et al. (120)	2017	42 ♀	Prospective, single-blinded, placebo controlled, randomized, parallel-group study, between subjects design	To evaluate the effectiveness of repeated tDCS over the occipital nerve (C2) and the DLPFC to reduce pain and fatigue in FMS.	Anodal tDCS & sham, 1.5 mA, 20 min bifrontal DLPFC (F3/F4), occipital nerve (C2), total of 8 sessions, 2 × for 4 weeks	-	NRS, PCS, MFIS	-	-	Laboratory	-	Eight sessions of bifrontal anodal tDCS over DLPFC resulted in pain and fatigue relief in FMS, whereas anodal tDCS of the occipital nerve only improved pain.
31	Villamar et al. (103)	2013	18 ♀	Patient- and assessor-blind, randomized, sham-controlled, crossover clinical trial with equal allocation ratio (1:1), within subjects design	To examine the effects of focal modulation of the left M1 with HD-tDCS in FMS.	Single sessions of anodal, cathodal, & sham tDCS over left M1, 2 mA, 20 min, with wahs-out period of 7 days to prevent carry-over effects	-	VNS for overall pain, VNS for anxiety, adapted QoL scale for persons with chronic illness, BDI-II,	-	PPT, Semmes-Weinstein Monofilaments (mechanical detection & pain)	Laboratory	-	Anodal & cathodal 4 × 1-ring HD-tDCS resulted in a significant reduction in overall perceived pain in FMS compared to sham stimulation.
32	Yoo et al. (101)	2018	58 ♀	Randomized, single-blind controlled trial, between sibjects design	To test the effect of combining anodal DLPFC and occipital nerve stimulation on FMS symptoms.	(1) tDCS on C2 (anode over right ON, cathode over left ON, 1.5 mA, 20 min); (2) tDCS on bilateral DLPFC before C2 stimulation (2 mA, 20 min, anodal tDCS F4, cathodal tDCS F3); (3) sham tDCS over ON; total of 8 sessions: 2 sessions/week, 4 weeks	-	FIQ, BDI, NRS	-	-	Laboratory	-	Adding right-anodal bifrontal tDCS to ONS did not have any added benefit in improving FMS related symptoms.

6-MWT, 6-minute-walking-test; BAI, beck anxiety inventory; BDI, beck depression inventory; BDNF, brain derivate neurotrophic factor; BDS, backward digit span; BFI, brief fatigue inventory; BOLD, blood-oxygen-level-dependent; BPI, brief pain inventory; CIRS, cumulative illness rating scale; COWAT, controlled oral word association test; CPT, cold pressure test; CSI-BP, central sensitization inventory-Brazilian population; CSP, cortical silent period; DASS, depression anxiety stress scale; FAS, fatigue assessment scale; FBDST, forward and backward digit span test; FDS, forward digit span; FIQ-R, fibromyalgia impact questionnaire-revised; FMI, Freiburg mindfulness inventory; fMRI, functional magnetic resonance imaging; FSQ, fibromyalgia survey questionnaire; HADS, hospital anxiety and depression scale; HAM, Hamilton anxiety scale; HAS, hypnotic analgesia suggestion; HPTh, heat pain threshold; HPTo, heat pain tolerance; McGill, McGill pain questionnaire; MEP, motor evoked potential; MFI, multidimensional fatigue inventory questionnaire; MFIS, modified fatigue impact scale; MINI, Minnesota international neuropsychiatric inventory; MMSE, mini mental state exam; MOSS-SS, medical outcomes study sleep scale; NRS, numeric rating scale; OCF, occipital nerve field; OIC, operculo insular cortex; ON, occipital nerve; PANAS, positive and negative affect scores; PASAT, paced auditory serial addition test; PCP:S, profile of chronic pain; PCS-B, pain catastrophizing scale-Brazilian version; PNS, numerical pain scale; POMS, profile of mood scale; PPT, pain pressure threshold; PSQI, Pittburgh sleep quality index; RAVLT, rey auditory verbal learning test; RMT, resting motor threshold; SCL-90R, symptom checklist 90 revised; SF36, quality of life questionnaire; SICI, short intercortical inhibition; SOPA-A, survey of pain attitudes-Brazilian population; SS, symptom severity scale; STAI-B, state trait anxiety inventory-Brazilian version; STAI-I, state-trait anxiety inventory I; STAI-II, Staite-trait anxiety inventory II; TAS, Toronto alexithymia scale; TMT, trail making test; VAS, visual analogue scale; VNS, visual numeric scale; WAIS, Wechsler adult intelligence test; WHOOOL, World Health Organization's quality of life questionnaire; WHYMPI, west Haven Yale multidimensional pain inventory; WMT, working memory training; WPI, widespread pain index.

**Table 2:** Significant symptom improvement of anodal (a-) or cathodal (c-) tDCS or tRNS vs. sham or control group on FMS (yes, +; no, −; NA, not assessed).

	Author	Year	*n*	Intervention	Pain	Sleep	Cognition	Depression	Anxiety	QoL	Functional performance
1	Arroyo-Fernández et al. (89)	2022	120 (113 ♀)	a-tDCS	+	NA	−	+	+	+	NA
2	Brietzke et al. (92)	2020	20 ♀	a-tDCS	+	+	+	+	−	−	NA
3	Castillo-Saavedra et al. (116)	2016	20 (17 ♀)	a-tDCS	+	−	−	−	−	+	−
4	Caumo et al. (107)	2022	48 ♀	a-tDCS	+	+	+	+	−	−	NA
5	Cummiford et al. (110)	2016	13 ♀	a-tDCS	+	NA	NA	NA	NA	NA	NA
6	Curatolo et al. (49)	2017	20 ♀	HF-tRNS	+	NA	+	+	+	+	NA
7	DallAgnol et al. (84)	2015	1 ♀	a-tDCS	+	NA	NA	−	+	NA	NA
8	de Melo et al. (111)	2020	31 ♀	a-tDCS	+	NA	NA	NA	−	NA	NA
9	Fagerlund et al. (117)	2015	48 (45 ♀)	a-tDCS	+	NA	NA	−	−	−	NA
10	Foerster et al. (109)	2015	12 ♀	a-tDCS	+	NA	NA	NA	NA	NA	NA
11	Forogh et al. (104)	2021	30 ♀	a-tDCS	+	NA	NA	+	+	+	NA
12	Jales et al. (118)	2015	20 ♀	a-tDCS	+	NA	NA	NA	NA	+	+
13	Kang et al. (55)	2020	46 (44 ♀)	a-tDCS	+	−	NA	+	−	+	NA
14	Khedr et al. (93)	2017	40 (34 ♀)	a-tDCS	+	NA	NA	+	+	NA	NA
15	Lim et al. (94)	2021	12 ♀	a-tDCS	+	NA	NA	NA	NA	NA	NA
16	Loreti et al. (95)	2023	35 ♀	a-tDCS	+	NA	NA	−	−	+	NA
17	Matias et al. (119)	2022	30 ♀	a-tDCS	+	NA	NA	NA	−	−	−
18	Matias et al. (99)	2022	47 ♀	a-tDCS	−	NA	NA	−	−	−	−
19	Mendonca et al. (98)	2016	45 ♀	a-tDCS	+	NA	NA	+	+	+	NA
20	Paula et al. (115)	2022	86 ♀	a-tDCS	+	NA	NA	+	+	+	NA
21	Ramasawmy et al. (100)	2022	30 (28 ♀)	a-tDCS	−	−	NA	−	−	+	NA
22	Rocca et al. (113)	2022	76 ♀	a-tDCS	NA	NA	NA	NA	NA	NA	NA
23	Samartin-Veiga et al. (106)	2022	130 ♀	a-tDCS	−	−	−	+	+	−	−
24	Samartin-Veiga et al. (102)	2022	130 ♀	a-tDCS	−	NA	NA	−	−	−	−
25	Santos et al. (108)	2018	40 ♀	a-tDCS	NA	NA	+	NA	NA	NA	NA
26	Schein et al. (112)	2023	18 ♀	a-tDCS	NA	NA	NA	NA	NA	NA	NA
27	Serrano et al. (105)	2022	36 ♀	a-tDCS	NA	NA	+	NA	NA	+	NA
28	Silva et al. (114)	2017	40 ♀	a-tDCS	+	NA	+	NA	NA	NA	NA
29	Terrasa et al. (see text footnote 1)	2025	39 ♀	a-tDCS	NA	NA	NA	NA	NA	NA	NA
30	To et al. (120)	2017	42 ♀	a-tDCS	+	NA	NA	NA	NA	NA	NA
31	Villamar et al. (103)	2013	18 ♀	a- & c- tDCS	+	NA	NA	−	NA	−	NA
32	Yoo et al. (101)	2018	58 ♀	a-tDCS	+	NA	NA	+	NA	+	NA

## Data extraction and transformation

### Change indices

To quantify stimulation-related effects on neurophysiological and neurochemical variables, we calculated a percentage change index (CI) for pre- and post-treatment values using the following formula:

ChangeIndex(CI)=((V2−V1)/V1))*100

Where *V*1 is the baseline and *V*2 the post-intervention value. This standardized approach allowed for comparability across studies using diverse outcome measures.

### Neurochemical and neurophysiological outcomes

#### BDNF

Brain-derived neurotrophic factor is a neurotrophin essential for neuronal survival, synaptic plasticity, and cognitive function (121–123). Altered BDNF levels in FMS have been linked to impaired neuroplasticity and central sensitization (124). Two studies reported divergent effects of anodal tDCS on serum BDNF levels. Caumo et al. (107) found modest increases in BDNF following active (2.25%) and sham (3.44%) tDCS. In contrast, Serrano et al. (105) observed a marked reduction after active stimulation (−36.62%) and a large increase after sham (+72.91%). Although both studies reported directionally positive effects, sham stimulation was associated with larger magnitude changes.

#### Alpha-2 power

Resting-state EEG studies in FMS reveal reduced alpha power, especially in frontal and parietal regions (125, 126). de Melo et al. (111) calculated alpha-2 power changes after 5 and 10 days of anodal tDCS. After 5 sessions, pain levels decreased by 27.86%, and alpha-2 power decreased across frontal (−122.43%), parietal (−46.95%), and occipital (−29.27%) regions. Following 10 sessions, the changes were smaller and mixed: pain (−8.74%), frontal (7.78%), parietal (8.47%), and occipital (3.28%). Sham stimulation also showed decreases in pain (−32.02%) and increases in alpha-2 power across all regions. These findings support altered thalamo-cortical rhythmicity and the concept of allostatic reference resetting (127, 128) as possible mechanisms in FMS.

#### Pressure pain threshold (PPT)

PPT reflects mechanical pain sensitivity and is a core diagnostic feature in FMS (129–133). Jales et al. (118) reported a 14.92% increase in PPT after anodal tDCS compared to 4.93% with sham, indicating decreased pain sensitivity following active stimulation.

#### Serum beta-endorphin

Beta-endorphin is an endogenous opioid peptide that modulates pain and mood through central and peripheral mechanisms (134). FMS patients often exhibit reduced beta-endorphin levels, contributing to impaired nociceptive regulation (135–137). Khedr et al. (93) found a 17.97% increase in beta-endorphin after anodal tDCS, supporting the involvement of the opioid system in tDCS-related analgesia.

#### Motor-evoked potentials (SICI, CSP)

Alterations in sensory processing within the motor cortex—such as heightened responses to tactile or painful stimuli—have been observed in fibromyalgia patients (138), potentially reflecting disrupted inhibitory control. These abnormalities can be evaluated through motor evoked potentials, particularly short intracortical inhibition (SICI) and cortical silent period (CSP), which are transcranial magnetic stimulation-derived markers of cortical inhibition often altered in chronic pain conditions (139–141). Notably, Schein et al. (112) reported that anodal tDCS significantly increased SICI and CSP by 36.99% and 18.13%, respectively—changes that may reflect enhanced GABAergic activity and a restoration of inhibitory motor network function (142–144).

#### Cognitive function

Cognitive dysfunction (“fibro fog”) in FMS includes impairments in attention, memory, and executive function (145–147). Serrano et al. (105) found that anodal tDCS significantly improved performance on the Trail Making Test A and B (TMT-A: −19.15%, TMT-B: −15.20%) and the Controlled Oral Word Association Test (orthographic: +9.92%, semantic: +16.49%), indicating enhancements in executive attention, processing speed, and verbal fluency.

#### Sensory gating

Sensory gating deficits in FMS result in impaired filtering of repetitive stimuli, contributing to sensory overload and chronic pain (148, 149). Terrasa et al. (see text footnote 1) reported that anodal tDCS improved sensory gating during late somatosensory processing stages. Gating effects increased by 126.72% in the ipsilateral hemisphere but decreased contralaterally, suggesting lateralized modulation of sensory filtering.

#### Hemodynamic variability (BOLD-fMRI)

BOLD-fMRI studies have shown altered cerebral perfusion and functional connectivity in FMS, particularly in pain-related and cognitive networks (150). Lim et al. (94) reported increased BOLD signal variability in the rostral anterior cingulate cortex, lateral prefrontal cortex, and thalamus following anodal tDCS. These changes correlated with pain reduction and improved network adaptability, supporting a neuromodulatory effect of tDCS on central pain processing.

### Summary neurochemical and neurophysiological outcomes

Studies examining changes in BDNF levels following tDCS report inconsistent findings. Caumo et al. (107) observed a moderate increase in BDNF after both active and sham tDCS conditions, whereas Serrano et al. (105) reported a marked decrease in BDNF following active stimulation. These discrepancies may be attributed to differences in electrode configurations, stimulation intensities, or patient characteristics such as baseline BDNF levels and disease severity.

This suggests that individual variability and protocol differences significantly influence tDCS effects on BDNF. Notably, the evidence indicates that active tDCS generally produces smaller increases in BDNF compared to sham, implying that its impact on this neuroplasticity marker may be less robust than initially hypothesized.

Regarding cortical activity, changes in alpha-2 power support the notion that anodal tDCS modulates neural activity within sensory processing and pain-related brain regions. A substantial reduction in alpha-2 power across frontal, parietal, and occipital regions was observed after 5 days of active tDCS, correlating with decreased pain perception. This suggests that tDCS may reset or modulate thalamo-cortical rhythmicity. However, after 10 days, these effects were less consistent, with smaller or even opposing changes in both pain levels and alpha-2 power, indicating a potential diminishing efficacy over time. Importantly, sham stimulation also induced changes in alpha-2 power and pain perception, underscoring the necessity for careful interpretation in sham-controlled trials.

Jales et al. (118) provided robust evidence that repetitive anodal tDCS enhances pressure pain thresholds in fibromyalgia syndrome (FMS) patients, supporting its role as a non-invasive analgesic intervention. Similarly, Khedr et al. (93) reported increased serum beta-endorphin levels post-tDCS, reinforcing the involvement of endogenous opioid release in mediating tDCS-induced analgesia.

From a neurophysiological perspective, Schein et al. (112) demonstrated enhanced intracortical inhibition following tDCS, evidenced by significant changes in short-interval intracortical inhibition and cortical silent period. These findings suggest that tDCS may improve motor cortex inhibitory mechanisms, which are critical for modulating motor symptoms and sensory processing deficits in FMS.

Cognitively, Serrano et al. (105) reported improvements in attention, memory, and processing speed following tDCS, which addresses the cognitive impairments commonly referred to as “fibro fog” in FMS patients. This indicates that tDCS may offer therapeutic benefits beyond pain relief, potentially enhancing patients' daily functioning and overall quality of life.

Additionally, Terrassa et al. (see text footnote 1) found that tDCS improved sensory gating, particularly during later stages of sensory information processing. This indicates that tDCS may facilitate better filtering of sensory input, reducing sensory overload and pain perception in FMS. Lim et al. (94) further corroborated these findings by demonstrating increased BOLD signal variability in key pain-related regions such as the rostral anterior cingulate cortex and thalamus, changes that correlated with pain reduction.

Therefore, the neurochemical and neurophysiological evidence indicates that tDCS exerts multifaceted effects in FMS patients. While its impact on BDNF levels remains inconclusive and may depend on individual and protocol-specific factors, tDCS consistently modulates cortical oscillations, enhances pain thresholds, and engages endogenous opioid pathways. Moreover, it improves intracortical inhibition and cognitive function, while facilitating sensory gating mechanisms. These findings highlight the complex, multi-level neural modulations induced by tDCS, supporting its therapeutic potential for both pain and cognitive symptoms in FMS. However, variability across studies underscores the need for further research to optimize stimulation protocols and elucidate underlying mechanisms (Table 3).

**Table 3:** Neurochemical and neurophysiological outcomes.

Neurochemical & neurophysiological variable	Study	tES protocol	% Change (CI)	Outcome
BDNF	Caumo et al. (2022) (107)	Anodal tDCS (left M1)	+2.5% (active) vs. +3.44% (sham)	Mixed results; anodal tDCS shows a smaller positive change compared to sham tDCS in BDNF levels.
	Serrano et al. (2022) (105)	Anodal tDCS (left M1)	−36.62% (active) vs. +72.91% (sham)	Large reduction in BDNF after active stimulation and significant increase after sham.
Alpha-2 power	de Melo et al. (111)	Anodal tDCS (5 and 10 days)	Pain −27.86% after 5 days vs. −8.74% after 10 days	Alpha-2 power decreases across brain regions after anodal tDCS treatment, showing a consistent trend of reduced pain and lower alpha-2 power.
			Frontal (−122.43%), Parietal (−46.95%), Occipital (−29.27%)	
			Sham tDCS: Pain (−32.02%) and Alpha-2 in frontal (+49.4%)	
PPT	Jales et al. 2015 (118)	Repetitive anodal tDCS (left M1)	+14.92% (active) vs. +4.93% (sham)	Repetitive anodal tDCS reduces pain sensitivity as measured by PPT.
Serum-beta endorphin	Khedr et al. (2017) (93)	Repetitive anodal tDCS (left M1)	+17.97% (active)	Significant increase in serum beta-endorphin levels after repetitive anodal tDCS.
Motor-evoked potentials	Schein et al. (2023) (112)	Anodal tDCS (left M1)	+ 36.99% (SICI) and +18.13% (CSP)	Improvement in motor cortex inhibition (SICI and CSP), indicating a potential enhancement in cortical excitability and inhibition.
Cognitive impairment	Serrano et al. (2022) (105)	Anodal tDCS (left M1)	TMT-A (−19.15%), TMT-B (−15.20%), COWAT (orthographic: +9.92%, semantic: +16.49%)	Improvement in executive functions, working memory, cognitive flexibility, and processing speed after anodal tDCS.
Sensory gating	Terrassa et al. (2025) (see text footnote 1)	Anodal tDCS (left S1)	Ipsilateral site: +126.72%; contralateral site: inverse effect	Enhanced sensory gating at later stages of information processing, with increased gating effect at the ipsilateral site.
BOLD/fMRI)	Lim et al. (2023) (94)	Anodal tDCS (left M1)	SD-BOLD: Increase in rACC, vmPFC, thalamus	Increased SD-BOLD in brain regions associated with pain processing and decreased pain perception after anodal tDCS.

BDNF, brain derived neurotrophic factor; PPT, pain pressure threshold; SD-BOLD, blood-oxygen-level-dependent signal; fMRI, functional magnetic resonance imaging; TMT, trail making test; COWAT, controlled oral word association test; SICI, short interval intracortica inhibition; rACC, rostral anterior cingular cortex; vmPFC, ventromedial prefrontal cortex; CSP, cortical silent period.

## Assessment of methodological heterogeneity

### Risk of bias assessment for RCTs

Risk of bias was independently assessed by two reviewers (CW and HC) using the Cochrane Risk of Bias tool and the PEDro scale. Inter-rater reliability was excellent, with a Cohen's kappa of *κ* = 0.9588, indicating strong agreement between raters. Of the 23 randomized controlled trials (RCTs) included, 56.52% (13/23) were rated as excellent quality, 34.78% (8/23) as good, and 8.70% (2/23) as fair (see Table 4).

**Table 4:** Risk of bias assessment of the RCTs (PEDro scale).

		C1	C2	C3	C4	C5	C6	C7	C8	C9	C10	C11	Total score
1	Arroyo-Fernández et al. (89)	+	+	+	+	+	+	+	+	+	+	+	10
2	Brietzke et al. (92)	+	+	+	+	+	+	+	+	+	+	+	10
3	Caumo et al. (107)	+	+	+	+	+	+	+	+	+	+	+	10
4	Samartin-Veiga et al. (106)	+	+	+	+	+	+	+	+	+	+	+	10
5	Samartin-Veiga et al. (102)	+	+	+	+	+	+	+	+	+	+	+	10
6	Serrano et al. (105)	+	+	+	+	+	+	+	+	+	+	+	10
7	Khedr et al. (93)	+	+	+	+	+	+	+	+	−	+	+	9
8	Loreti et al. (95)	+	+	+	+	+	+	+	+	−	+	+	9
9	Matias et al. (119)	+	+	+	+	+	+	+	+	−	+	+	9
10	Matias et al. (99)	+	+	+	+	+	+	+	+	−	+	+	9
11	Mendonca et al. (98)	+	+	+	+	+	+	+	+	−	+	+	9
12	Paula et al. (115)	+	+	+	+	+	+	+	+	−	+	+	9
13	Santos et al. (108)	+	+	+	+	+	+	+	+	−	+	+	9
14	de Melo et al. (111)	+	+	+	+	+	−	+	+	−	+	+	8
15	Fagerlund et al. (117)	+	+	+	+	+	+	−	+	−	+	+	8
16	Jales et al. (118)	+	+	?	?	+	+	+	+	+	+	+	8
17	Ramasawmy et al. (100)	+	+	+	?	+	+	+	+	−	+	+	8
18	Terrasa et al. i.r.	+	+	+	+	+	+	−	+	−	+	+	8
19	Forogh et al. (104)	+	+	?	+	−	−	+	+	+	+	+	7
20	To et al. (120)	+	+	+	?	+	−	−	+	+	+	+	7
21	Yoo et al. (101)	+	+	+	−	−	−	+	+	+	+	+	7
22	Curatolo et al. (49)	+	+	?	+	?	?	?	+	?	+	+	5
23	Rocca et al. (113)	+	+	−	−	−	−	−	+	−	+	+	4

▪, high rob; ▪, some concerns; ▪, low rob; rob, risk of bias; C1, eligibility criteria were specified; C2, random allocation of subjects; C3, concealed allocation; C4, no baseline differences; C5, blinding of subjects; C6, blinding of therapist; C7, blinding of assessors; C8, measures obtained from more than 85% of subjects; C9, ITT analyses; C10, at least one key outcome of between-group comparison reported; C11, variablity and point measures for at least one key outcome. For more information, see Appendix or PEDro scale (151). http://www.pedro.org.au/wp-content/uploads/PEDro_scale.pdf.

Low risk of bias was commonly associated with domains such as clearly defined eligibility criteria, random and concealed allocation, appropriate outcome measurement, between-group statistical comparisons, effect size estimation, and variability reporting. Conversely, unclear or high risk of bias frequently occurred in relation to baseline group similarity, and blinding of participants, therapists, and outcome assessors.

Notably, 52% of the RCTs did not perform an intention-to-treat analysis (ITT), indicating that approximately half of the trials failed to analyze participants in the groups to which they were randomized, regardless of adherence to the intervention (152). Intention-to-treat analysis thus emerged as the most frequently neglected source of bias.

While PEDro scoring categories are commonly used to summarize study quality (“excellent,” “good,” “fair”), it is important to note that these global ratings may obscure high risk of bias in specific methodological domains. For instance, the two studies rated as “fair” based on total PEDro scores still exhibited high risk of bias across several individual criteria.

Low risk of bias was associated with several factors, including:

▪ Clear eligibility criteria
▪ Random and concealed allocation of participants
▪ Accurate measurement of outcomes
▪ Between-group statistical comparisons
▪ Effect size reporting
▪ Reporting of variability measures

Conversely, high or unclear risks of bias were linked to the following areas:

▪ Similarity of groups at baseline
▪ Blinding of participants, therapists administering treatment, and assessors measuring key outcomes

A notable concern is that 52% of the randomized controlled trials did not perform ITT analysis, indicating that approximately half of the studies excluded some randomized participants from the final analysis based on intervention adherence. Since ITT analysis is essential for minimizing bias and preserving the integrity of randomized allocation, its omission represents the most frequently neglected or violated risk of bias criterion in this review (152).

### Risk of bias assessment for crossover trials

Risk of bias in the six crossover trials was assessed using an adapted version of the Cochrane Collaboration's risk-of-bias tool (87). Notably, three studies (50%) failed to report on important methodological details such as appropriate design, carry-over effects, allocation concealment, blinding, or incomplete outcome data, leaving these factors unclear. For these studies, high risk of bias was linked to issues such as insufficient washout periods, lack of randomization, and failure to blind participants. In the context of tDCS, a washout period of 7–14 days (with a mean of 9.9 days) might have been insufficient, potentially leading to carry-over effects. On the other hand, the remaining three studies exhibited excellent methodological quality, demonstrating a low risk of bias (Table 5).

**Table 5:** Risk of bias and quality assessment for cross-over trials (cochrane quality assessment).

		1	2	3	4	5	6	7	8	9	
1	Cummiford et al. (110)	?	−	?	+	?	?	−	?	?	Multiple sessions
2	Foerster et al. (109)	?	−	?	?	?	?	?	?	?	Multiple sessions
3	Lim et al. (94)	?	−	?	?	?	?	?	?	?	Multiple sessions
4	Schein et al. (112)	+	+	+	+	+	+	+	+	+	Single session
5	Silva et al. (114)	+	+	+	+	+	+	+	+	+	Single session
6	Villamar et al. (103)	+	+	+	+	+	+	+	+	+	Single session

1, appropriate cross-over design; 2, randomized treatment order; 3, carry-over effect; 4, unbiased data; 5, allocation concealment; 6, blinding; 7, incomplete outcome data; 8, selective outcome reporting; 9, other bias; for more information: https://handbook-5-1.cochrane.org/chapter_8/8_assessing_risk_of_bias_in_included_studies.htm.

### Risk of bias assessment for single-arm studies

Risk of bias for the single-arm studies was evaluated using the Cochrane Collaboration's MINORS checklist (153). To be deemed eligible, a study must score at least 13 out of 16 points (153, 154). Both studies in this category achieved 14 points, indicating a low risk of bias. Each met all criteria except for blinding of endpoint assessment (see Table 6).

**Table 6:** Risk of bias and quality assessment for single-arm studies (MINORS ROB, 153).

		1	2	3	4	5	6	7	8	Total score
1	Castillo-Saavedra et al. (116)	2	2	2	2	0	2	2	2	14
2	Kang et al. (55)	2	2	2	2	0	2	2	2	14

0, not reported; 1, reported but inadequate; 2, reported and adequate; max. 16 points for non-comparative studies.

### Risk of bias assessment for the single-case study

The quality of the single case study was assessed using a risk-of-bias tool developed by Reichow et al. (83). This tool uses an eight-item scale, with each item judged as either “criteria fulfilled,” “criteria not fulfilled,” or “unclear.” For the case study reviewed, all criteria were clearly fulfilled except for (Table 7):

▪ Item 3: Blinding of participant and personnel
▪ Item 5: Blinding of outcome reporting.

**Table 7:** Risk of bias assessment for the single-case study.

		1	2	3	4	5	6	7	8	Total score
1	DallAgno et al. (84)	+	+	?	+	?	+	+	+	6

1, sequence generation (selection bias); 2, participant selection (selection bias); blinding of participants and personnel (performance bias); 4, procedural fidelity (performance bias); 5, blinding of outcome assessment (detection bias); 6, selective outcome reporting (detection bias); 7, dependent variable reliability (detection bias); 8, data sampling (detection bias). Quality scores: 10–9: excellent; 8–6: good; 5–4: fair; 3–0: poor (155).

## Discussion

This systematic review and meta-analysis set out to evaluate the clinical, neurophysiological, and neurochemical effects of non-invasive brain stimulation, particularly tDCS, in individuals with FMS. Overall, the findings suggest that anodal tDCS offers promising short-term benefits for pain relief, mood, and certain cognitive symptoms. However, several key factors—such as protocol duration, stimulation site, patient characteristics, and methodological quality—influence outcomes and must be taken into account when interpreting these effects.

### Clinical outcomes

Across the included studies, a clear pattern emerged: 89% reported significant pain reductions following tDCS, especially with stimulation over M1 and DLPFC Both sites effectively increased pain thresholds and tolerance, likely due to the diffuse, non-focal nature of standard tDCS protocols. This broad effect offers clinical flexibility to tailor treatments based on symptom profiles but complicates understanding specific neural mechanisms. Consequently, more focal methods—such as high-definition tDCS or image-guided repetitive transcranial magnetic stimulation—are needed to clarify site-specific effects.

Session duration and scheduling were also critical for treatment success. Protocols lasting at least 4 weeks consistently produced stronger, longer-lasting effects, indicating a time-dependent accumulation of neuroplastic changes. This highlights that not only the number but also the spacing of sessions significantly influences tDCS outcomes. Moreover, timing relative to task performance or injury further modulates the intervention's efficacy on motor and sensory functions.

For instance, in healthy individuals, applying tDCS prior to motor training has been shown to enhance motor skill learning (156). The benefits of pre-training stimulation suggest that tDCS might facilitate neuroplastic changes, which could optimize the brain's response to subsequent motor training (156).

In the context of neurorehabilitation, particularly stroke recovery, the timing of tDCS relative to therapeutic interventions has shown differential effects. When tDCS was applied sequentially—prior to therapy—it significantly improved daily functioning and movement efficiency, especially in the paretic hand, compared to concurrent or sham stimulation (157). This indicates that time-dependent neuroplastic changes induced by tDCS may be most advantageous when administered before the intervention, facilitating enhanced motor control and functional recovery.

Similarly, animal models of chronic neuropathic pain demonstrated that the timing of tDCS post-injury influences its efficacy. Early application of tDCS not only prevented pain onset but also produced longer-lasting analgesic effects (158). Additionally, more frequent stimulation sessions yielded greater pain relief, with comparable outcomes observed for both ipsilateral and contralateral M1 stimulation. These findings support the hypothesis that early and frequent tDCS interventions may optimize therapeutic outcomes in pain management.

This systematic review further revealed that longer-duration interventions—particularly targeting M1—resulted in the most significant improvements in both pain and mood; however, these benefits typically diminished following treatment cessation. Therefore, future research should investigate strategies to sustain these effects over time, potentially through booster sessions or adjunct therapies.

Combination treatments yielded mixed results. While pairing tDCS with aerobic exercise showed benefits in some trials, other combinations—such as with mindfulness or functional training—did not consistently enhance outcomes. These inconsistencies likely arise from variability in individual responsiveness, protocol design, and intervention timing. Thus, not all combination approaches are equally effective, underscoring the need for more nuanced research to identify synergistic strategies.

While these findings point to potential therapeutic effects, it is important to distinguish between statistically significant outcomes and clinically meaningful improvements. As highlighted by Willigenburg and Poolman (159), clinical interpretation requires more than just *p*-values—it depends on patient-centered metrics such as perceived benefit and meaningful change. Our analysis found that although tDCS was associated with statistically significant reductions in pain, the observed effect sizes were small (Hedge's *g* < 0.2) (see Supplement 7, Appendix) and none of the included studies reported Minimal Clinically Important Differences or comparable thresholds. This limits the ability to judge the practical impact of these findings, and underscores the need for future trials to include such benchmarks in evaluating treatment efficacy.

### Objective and subjective pain measures

Across the reviewed studies, pain reduction was assessed using both subjective and objective measures, providing a multidimensional view of treatment response. Most trials relied on self-reported pain ratings (e.g., VAS or NRS), which reflect the personal and psychological experience of pain but may vary with mood, expectation, or contextual factors. These subjective outcomes were complemented by more objective indices, such as pressure pain threshold, neurophysiological markers (e.g., somatosensory evoked potentials), and neurochemical measures (e.g., β-endorphin levels, Glx concentrations). For example Jales et al. (118), reported a 14.92% increase in pain pressure threshold following anodal tDCS, indicating reduced mechanical pain sensitivity, while Khedr et al. (93) observed a 17.97% increase in serum β-endorphins, a change positively associated with pain relief. This convergence between subjective and objective measures suggests that anodal tDCS likely acts through both sensory-discriminative and affective-motivational pain systems, which are commonly disrupted in FMS.

### Clinical relevance and minimal clinically important difference (MCID)

While statistical significance was consistently reported across studies, clinical significance—whether the observed changes are meaningful to patients—also deserves attention. The MCID represents the smallest change in a symptom that patients perceive as beneficial (160). For chronic pain conditions such as FMS, the MCID for pain is estimated to be 14% on the Fibromyalgia Impact Questionnaire total score (161), around 30%–35% improvement on the Brief Pain Inventory (162), a reduction of aproximately 30% on the pain intensity-NRS for a moderate clinically important change (163).

However, there is significant variability in how the MCID is defined and applied across studies. A recent systematic review highlighted considerable heterogeneity in MCID estimates for chronic pain, with absolute MCID values ranging from 12 to 39 mm on a 0–100 mm VAS scale, and relative MCID values varying between 22% and 45% (164). The review also emphasized that baseline pain levels were strongly associated with the MCID, suggesting that patients with higher baseline pain may experience more meaningful treatment effects (164). Unfortunately, none of the studies included in this review explicitly reported whether their findings met MCID thresholds, which is a critical gap in the literature. Given this, future research should incorporate MCID-based assessments to better determine whether observed improvements in pain, mood, and cognitive function following tDCS are truly meaningful for patients.

### Neurophysiological and neurochemical mechanisms

Neurophysiological findings from 13 studies provided further insight into how tDCS might exert its effects in FMS. Changes in BDNF, Glx, β-endorphins, and cortical excitability markers point toward a neuromodulatory mechanism rooted in plasticity, opioid release, and altered cortical inhibition. For instance, increased β-endorphins following M1 stimulation were correlated with improved pain thresholds, while decreases in Glx in the ACC were linked to reduced central sensitization. However, BDNF results were inconsistent across studies, suggesting individual differences in baseline neuroplastic potential may moderate outcomes.

Functional connectivity and cortical dynamics also changed following stimulation. Altered thalamocortical connectivity, changes in BOLD variability, and modulation of cortical oscillations support the idea that tDCS can reorganize dysfunctional networks in FMS. Still, the clinical significance of these findings remains uncertain, as not all neurophysiological changes mapped directly onto symptom improvement.

Similarly, studies on cortical excitability and sensory gating showed that tDCS can modulate inhibitory control and somatosensory filtering, both of which are known to be impaired in FMS. While these results are preliminary, they provide a compelling rationale for further exploration of biomarkers as predictors of treatment response.

To better understand the effects of tDCS, it is important to consider current research on brain networks and neuroinflammation, particularly its potential relevance for conditions like FMS. For instance, an exploratory study in patients with disorders of consciousness following traumatic brain injury demonstrated that tDCS could enhance cortical activation and induce anti-inflammatory effects, which were linked to behavioral improvements (165). Specifically, multiple tDCS sessions reduced circulating inflammatory markers, such as angiopoietin-2, vascular endothelial growth factor C, and interferon gamma-induced protein 10 (165), which are also implicated in chronic pain conditions like FMS (166, 167). Given that FMS is associated with central sensitization and neuroinflammation, these findings suggest that tDCS may offer similar neurophysiological benefits in FMS, improving cortical activation and potentially alleviating neuroinflammatory processes involved in pain perception and central sensitization. Although FMS has traditionally been considered a non-inflammatory disorder, emerging research suggests that low-grade inflammation in the central nervous system—referred to as neuroinflammation—may play a significant role in its pathophysiology. Studies have shown microglial activation and increased levels of pro-inflammatory cytokines within the brain and cerebrospinal fluid of individuals with FMS (168). This neuroinflammatory response is believed to contribute to central sensitization, a process in which the central nervous system becomes hypersensitive to stimuli, thereby amplifying pain and other sensory experiences in FMS (169). Further evidence suggests that inflammatory cytokines, such as IL-6 and IL-8, and microglial activation are key contributors to the pathogenesis of FMS (170).

### Cognitive and affective effects

Cognitive impairments are a major concern for many FMS patients, and tDCS—particularly when combined with cognitive training—has shown potential in addressing this. Improvements in executive function, memory, and attention were reported in several studies, especially when targeting the DLPFC. These cognitive gains were often moderated by BDNF levels, reinforcing the idea that tDCS interacts with the brain's plasticity systems. However, improvements in overall quality of life were less consistent, possibly due to the multifaceted nature of this construct and variability in measurement approaches.

### Optimal treatment location, duration, and intensity

Based on the studies included in this systematic review, the optimal treatment location(s) for tDCS generally target brain areas involved in pain processing and motor control. Specifically, the M1 and DLPFC are most commonly targeted in FMS and other chronic pain conditions. Neurobiologically, the DLPFC is thought to influence the affective and cognitive dimensions of pain, such as emotional regulation and pain perception, via its role in executive function and top-down modulation (171). The DLPFC is involved in cognitive and emotional aspects of pain and shows structural and functional changes in chronic pain conditions (171). It plays a role in both pain perception and suppression, with noninvasive stimulation of the left DLPFC showing potential to alleviate chronic pain by modulating brain networks related to pain and emotion (171). On the other side, stimulation of M1, especially over the anterior bank of the central sulcus corresponding to the painful area, has shown to produce significant pain relief, unlike stimulation of adjacent brain regions (172). These distinct mechanisms may explain differences in clinical outcomes observed across studies. However, the exact location may vary depending on the specific pathology and symptoms of the condition being treated.

In terms of intensity, the typical current intensity ranged from 1 to 2 mA, with 1.5 mA being the most commonly used in studies. However, the optimal intensity may vary depending on the individual's tolerance and the condition being treated. Higher intensities (e.g., 2 mA) might yield more pronounced effects, though they could also lead to increased side effects. Thus, individual adjustments and careful monitoring are essential.

Regarding treatment duration, the most frequently used protocol across studies in this review involved five sessions per week for 2 weeks, with each session lasting around 20–40 min. Some studies suggest that longer treatment periods, extending up to 4 weeks, may provide more sustained benefits, though the evidence remains mixed. Notably, Teixeira et al. (60) found that tDCS protocols lasting 4 weeks or more yielded larger effect sizes than shorter protocols. These results persisted even after adjusting for the total number of sessions, underscoring the importance of session distribution across weeks rather than simply the overall session count. In their longitudinal analysis, the most significant clinical improvements in FMS patients were seen after 4 weeks of stimulation (approximately 15 sessions), suggesting a time-dependent aspect to tDCS effects in FMS treatment.

Teixeira et al. (60) also highlight the physiological underpinnings of time-dependent effects, suggesting that neuroplasticity, particularly the N-Methyl-D-Aspartate-related networks in the brain, plays a critical role in the cumulative effectiveness of tDCS. The duration and timing of stimulation influence the magnitude and sustainability of plasticity changes in the brain. Long intervals between stimulation sessions might facilitate homeostatic plasticity, allowing for the stabilization and cumulative gain of neuroplastic effects. This could explain why longer stimulation durations result in more pronounced and sustained benefits in FMS, particularly for pain management.

Overall, while shorter durations of tDCS treatment may provide immediate relief, evidence suggests that longer protocols may result in more sustained improvements, especially in chronic conditions like fibromyalgia. The potential for cumulative effects emphasizes the importance of understanding the time-dependency of tDCS in optimizing its clinical application for FMS.

### Placebo effect

The placebo effect is a notable consideration in neuromodulation treatments, particularly in studies with FMS patients. For instance, some studies have reported significant improvements in pain, fatigue, and cognitive symptoms following sham tDCS (93, 102, 106). This may be explained by psychosocial factors or the release of endogenous opioids triggered by sham stimulation (173). Additionally, studies like To et al. (120) showed that anodal tDCS of the occipital nerve reduced pain but had no effect on fatigue. Similarly, a single session of anodal tDCS improved sensory gating in FMS patients (see text footnote 1).

### Implications for home management of FMS

There is growing interest in home-based tDCS as a potential adjunctive intervention for FMS. This approach offers practical advantages such as accessibility and reduced treatment burden, particularly for individuals facing physical, logistical, or financial barriers to frequent in-clinic sessions.

Preliminary studies have explored its feasibility and potential utility in related conditions, including major depressive disorder, anxiety, and chronic pain (92, 107, 174). In the context of FMS, recent trials suggest that home-based tDCS, when guided by clinicians and supported with appropriate training, may help modulate pain-related brain circuits and offer symptom relief (175, 176). However, these findings remain exploratory, and evidence for its effectiveness in FMS is not yet conclusive. While home-based tDCS is generally regarded as safe and well-tolerated in controlled settings, its use outside supervised environments introduces specific risks. These include potential misuse, inconsistent adherence, and improper electrode placement. Adverse events, although typically mild (e.g., skin irritation), must still be carefully monitored, and robust patient education is essential to minimize risk (177). Encouragingly, some studies have reported high adherence rates (e.g., 178), but real-world data remain limited.

Therefore, although home-based tDCS may represent a promising future direction for FMS management, it should currently be considered investigational. It must be applied cautiously, under professional supervision, and supported by rigorous research evaluating long-term safety, clinical efficacy, and implementation strategies. Future studies should focus on identifying patient selection criteria, optimal stimulation protocols, and integrating remote monitoring to ensure safe and effective use in home environments.

### Adverse effects

#### Somatosensory adverse effects

The most frequently reported adverse effects across studies in this systematic review were tingling, itching, and burning sensations. These symptoms were observed in both active and sham tDCS groups, suggesting they are mild, nonspecific, and generally well tolerated. Tingling was commonly reported by Castillo-Saavedra et al. (116), Matias et al. (119), and Serrano et al. (105); itching by Matias, et al. (99), and Villamar et al. (103); and burning sensations by Caumo et al. (107). These effects were typically transient and rarely led to treatment discontinuation.

Interestingly, Delicado-Miralles et al. (179) noted that somatosensory symptoms, including itching and neck pain, were more consistently associated with active tDCS sessions and remained stable over repeated treatments. In contrast, these symptoms decreased over time in the sham group, likely due to habituation. This pattern may compromise blinding, as participants could infer treatment allocation based on the presence or absence of such sensations—a critical consideration for trial design.

In this line, Rahimibarghani and Fateh (180) described a case in which a FMS patient experienced mild irritability, agitation, and headaches following tDCS. These effects were transient and resolved quickly, consistent with the general observation that somatosensory adverse effects are typically mild in this population. Nonetheless, the possibility of mood-related effects warrants further investigation.

In summary, tingling, itching, and burning are common somatosensory adverse effects in tDCS studies but are generally mild and short-lived. Their differing trajectories in active versus sham groups underlines the importance of careful monitoring and reporting, particularly to preserve blinding and ensure methodological rigor in future research.

#### Mood-related adverse effects

While mild somatosensory adverse effects are the most common, mood-related side effects, although less frequent, have also been reported and deserve closer attention. Studies in this sytsematic review, including Fagerlund et al. (117) and Caumo et al. (107), highlighted that mood changes, such as irritability, anxiety, and emotional instability, were more commonly observed following active tDCS treatment. These effects were typically mild and did not lead to treatment discontinuation. However, the case reported by Rahimibarghani and Fateh (180) provides a more concerning example, where a FMS patient's mood deteriorated significantly after a single tDCS session. Initially, the patient experienced irritability and agitation, but these symptoms escalated over the next 2 months into more severe mood disturbances, including anxiety, verbal aggression, poor sleep, and concentration issues. Although these symptoms did not lead to a psychiatric crisis, they raise important concerns about the potential for tDCS to trigger or exacerbate underlying mood disorders, particularly in individuals with preexisting psychological vulnerabilities.

The findings by Delicado-Miralles et al. (179) support the idea that mood disturbances could be a side effect of tDCS, as they noted a direct link between specific adverse effects (like neck pain) and active tDCS, which could indicate a broader impact of brain stimulation on mood and emotional regulation. Additionally, the long-term nature of mood changes reported by Rahimibarghani and Fateh (180) suggests that tDCS may have lingering effects that are not immediately apparent but may become more problematic over time, especially in patients with complex health profiles like FMS.

These mood-related adverse effects are particularly significant in the context of FMS, where patients often have comorbid psychiatric conditions, including depression and anxiety, which could be exacerbated by the stimulation. It's essential to consider the potential for these effects when treating FMS patients with tDCS, especially if they have a history of mood instability.

#### Headaches and other moderate adverse effects

Headaches were another common adverse effect, though generally mild in nature. Several studies, including those by Brietzke et al. (92) and Mendonca et al. (98), reported headaches as a mild side effect, primarily in the active stimulation groups. While these headaches did not typically interfere with the continuation of treatment, their consistent appearance suggests that they may be a predictable response to tDCS. Similarly, neck pain was noted in some studies, such as by Caumo et al. (107), but like headaches, this symptom was mild and not significant enough to affect treatment adherence. These moderate adverse effects were less frequent than the somatosensory effects but still noteworthy as they could represent potential barriers to treatment continuity for some patients.

Delicado-Miralles et al. (179) reported that neck pain occurred exclusively in the active tDCS group, suggesting that certain adverse effects, particularly those involving the neck and upper body, may be directly related to stimulation. Although generally mild, these effects were more frequent with active tDCS, which point to their relevance for protocol design.

#### Fatigue and dizziness

Fatigue and dizziness, while less commonly reported, did appear in some studies (e.g., 99). These side effects were generally short-lived and did not lead to significant adverse outcomes. However, these effects highlight the broader range of potential responses to tDCS, and while they are not as prevalent as somatosensory or mood-related adverse effects, they still warrant attention in clinical settings.

To summarize, the adverse effects of tDCS in FMS patients, as discussed in the systematic review and supplemented by the findings of Rahimibarghani and Fateh (180) and Delicado-Miralles et al. (179), can be grouped into somatosensory and mood-related effects (e.g., headache or increased anxiety). The most common and mild adverse effects include tingling, itching, burning, and skin redness, which are generally well-tolerated and transient. Moderate adverse effects such as headaches, neck pain, and mood changes appear less frequently but still merit attention, particularly in patients with complex health profiles. The evolution of mood-related side effects, as seen in the case of Rahimibarghani and Fateh (180), suggests that some individuals may experience longer-term mood changes, raising concerns about the potential for tDCS to exacerbate preexisting psychiatric conditions. Overall, these findings reinforce the need for careful patient selection and ongoing monitoring when using tDCS in FMS treatment, particularly for individuals with comorbid mood disorders.

#### Methodological considerations

The overall methodological quality of the included studies was reasonably high. Most RCTs were rated as excellent or good, and the risk of bias was generally low in the single-arm and crossover trials that provided sufficient methodological detail. However, a major limitation was the widespread omission of ITT analysis—reported in only 48% of RCTs—which may inflate effect sizes or introduce bias. Recurrent issues also included inadequate blinding and lack of baseline group similarity, particularly in crossover and case studies. These methodological concerns should be taken into account when interpreting the evidence base, as they point to the need for more rigorous trial designs in future research. Additionally, relying solely on total PEDro scores to classify study quality of RCTs may oversimplify underlying risks, as studies rated “fair” may still exhibit high bias in multiple domains, potentially undermining the robustness of their findings.

#### Summary of meta-analytic findings

Of the six outcomes analyzed, only pain intensity showed a statistically significant effect of anodal tDCS. However, the very small effect size highlights the need for clinical benchmarks, such as the MCID, to determine its practical relevance. No significant effects were observed for other variables (see Supplement 7, Appendix).

Overall, this systematic review and meta-analysis suggests that anodal tDCS—particularly when applied over several weeks—can produce meaningful short- and mid-term improvements in pain, mood, and cognitive function in FMS. These effects appear to be mediated by changes in neuroplasticity, cortical excitability, and functional connectivity, although outcomes vary across individuals and studies. Adverse effects were generally mild, with the most common being tingling, itching, and headaches, though some mood disturbances were also reported.

For clinical implementation, flexible yet structured protocols that account for individual differences and integrate targeted behavioral strategies may maximize benefits. Despite strengths such as the inclusion of high-quality RCTs, limitations include a lack of standardized treatment protocols and insufficient long-term follow-up data. Future research should emphasize personalized treatment approaches, longer intervention durations, and multimodal strategies to optimize tDCS efficacy in FMS management.

It is important not to overstate the efficacy of tDCS based on current evidence. Consistent with the evidence-based guidelines by Lefaucheur et al. (181), anodal tDCS targeting the left M1 with a right orbitofrontal cathode carries a Level B recommendation for FMS, indicating probable efficacy. Notably, no applications of tDCS in FMS or related conditions have yet achieved a Level A recommendation, which shows the absence of definitive high-quality evidence.

Our review aligns with this cautious classification. While most studies report statistically significant pain reductions following tDCS, the associated neurobiological changes—such as increased β-endorphins or decreased Glx—are inconsistently linked to clinical improvements, and no reliable biomarker has been validated to date. Mechanistic understanding remains limited, particularly regarding the modulation of large-scale neural networks, which are hypothesized to play a central role in symptom expression and regulation in FMS.

Current research predominantly focuses on localized brain areas, especially M1 and DLPFC. Although emerging studies have examined resting-state functional connectivity and neural oscillations (54, 182), they have yet to provide a comprehensive understanding of how tDCS influences large-scale brain networks in FMS. Addressing this gap is critical to advancing both mechanistic insights and clinical applications.

In conclusion, while tDCS shows promise as a non-invasive intervention for FMS, its clinical benefits should be interpreted cautiously. The current scientific evidence calls for further rigorous, large-scale studies, particularly those incorporating multimodal neuroimaging to clarify network-level effects and validate potential biomarkers. Only through such research can more definitive conclusions and stronger clinical recommendations be established.

## Limitations and future directions

Several limitations of the current work should be noted. First, the heterogeneity in stimulation protocols, targeted brain areas, outcome measures, and participant characteristics complicates direct comparisons across studies. Second, many studies featured small sample sizes and brief follow-up periods, which limits conclusions about the long-term efficacy of tDCS. Third, while some neurophysiological findings appear promising, they are preliminary and require replication in larger, well-controlled, multimodal studies. Fourth, although widely used in the literature for the assessment of the methodological quality of randomized controlled trials, the descriptive labels applied to the PEDro scale total scores (e.g., “excellent,” “good,” “fair”) are not formally validated. A total score in the “fair” range may still reflect high risk of bias in multiple individual domains. This limitation should be considered when interpreting the methodological quality of the included studies.

Future research should focus on:

• Investigating potential biomarkers and symptom profiles to inform personalized stimulation parameters
• Extending treatment durations and evaluating strategies for maintaining therapeutic effects
• Combining tDCS with behavioral or pharmacological interventions in rigorously controlled designs
• Exploring more focal stimulation methods such as high-definition tDCS
• Incorporating multimodal imaging and electrophysiology to better link neural changes with clinical outcomes
• Evaluating clinical relevance, including MCIDs.

## Conclusion

In conclusion, tDCS demonstrates potential benefits for pain reduction in FMS, supported by probable efficacy according to current guidelines. However, the evidence remains preliminary, with inconsistent neurobiological findings and a lack of validated biomarkers. The limited understanding of its effects on large-scale brain networks further highlights the need for more comprehensive research. Future studies should focus on elucidating the neural mechanisms of tDCS and refining protocols to optimize clinical outcomes.

## Data Availability

The original contributions presented in the study are included in the article/Supplementary Material, further inquiries can be directed to the corresponding author.

## Footnotes

^1^Terrasa J, Winterholler C, Montoya P, Juan A, Montoro C. Modulatory effects of non-invasive transcranial direct current stimulation on tactile stimulation processing in patients with Fibromyalgia Syndrome (2025); [Manuscript submitted for publication].

## References

[1] Bhargava J Hurley JA. Fibromyalgia. In: StatPearls. Treasure Island (FL): StatPearls Publishing (2025). Available online at: https://www.ncbi.nlm.nih.gov/books/NBK540974/

[2] Clauw DJ. Fibromyalgia: a clinical review. J Am Med Assoc. (2014) 311(15):1547–55. 10.1001/jama.2014.3266 24737367

[3] Nicholas M Vlaeyen JWS Rief W Barke A Aziz Q Benoliel R The IASP classification of chronic pain for ICD-11: chronic primary pain. Pain. (2019) 160(1):28–37. 10.1097/j.pain.0000000000001390 30586068

[4] Pomares FB Funck T Feier NA Roy S Daigle-Martel A Ceko M Histological underpinnings of grey matter changes in fibromyalgia investigated using multimodal brain imaging. J Neurosci. (2017) 37(5):1090–101. 10.1523/JNEUROSCI.2619-16.2016 27986927 PMC6596849

[5] Ruschak I Montesó-Curto P Rosselló L Aguilar Martín C Sánchez-Montesó L Toussaint L. Fibromyalgia syndrome pain in men and women: a scoping review. Healthcare. (2023) 11(2):223. 10.3390/healthcare11020223 36673591 PMC9859454

[6] Marques AP Santo ASDE Berssaneti AA Matsutani LA Yuan SLK. Prevalence of fibromyalgia: literature review update. Rev Bras Reumatol. (2017) 57(4):356–63. 10.1016/j.rbre.2017.01.005 28743363

[7] Amris K Ibsen R Duhn PH Olsen J Lolk K Kjellberg J Health inequities and societal costs for patients with fibromyalgia and their spouses: a Danish cohort study. RMD Open. (2024) 10(1):e003904. 10.1136/rmdopen-2023-003904 38307700 PMC10840036

[8] Ghio GA Jaen Manzanera A Torguet Carbonell J Donoso Isla CI Falcón Marchena AJ Martínez Pardo S. Health personnel perception about central sensitivity syndrome-fibromyalgia patients. Reumatol Clin. (2024) 20(2):73–9. 10.1016/j.reumae.2024.01.004 38342740

[9] Otón T Carmona L Rivera J. Patient-journey of fibromyalgia patients: a scoping review. Reumatol Clin. (2024) 20(2):96–103. 10.1016/j.reumae.2023.07.005 38395498

[10] Al Sharie S Varga SJ Al-Husinat L Sarzi-Puttini P Araydah M Bal'awi BR Unraveling the complex web of fibromyalgia: a narrative review. Medicina. (2024) 60(2):272. 10.3390/medicina60020272 38399559 PMC10890445

[11] Alda M Luciano JV Andrés E Serrano-Blanco A Rodero B del Hoyo YL Effectiveness of cognitive behaviour therapy for the treatment of catastrophisation in patients with fibromyalgia: a randomised controlled trial. Arthritis Res Ther. (2011) 13(5):R173. 10.1186/ar3496 22018333 PMC3308108

[12] Algar-Ramírez M Úbeda-D'Ocasar E Hervás-Pérez JP. Efficacy of manual lymph drainage and myofascial therapy in patients with fibromyalgia: a systematic review (Wirksamkeit manueller Lymphdrainage und myofaszialer Therapie bei Patienten mit Fibromyalgie: Ein systematischer Überblick). Schmerz. (2021) 35(5):349–59. 10.1007/s00482-020-00520-7 33326048

[13] Alorfi NM. Pharmacological treatments of fibromyalgia in adults; overview of phase IV clinical trials. Front Pharmacol. (2022) 13:1017129. 10.3389/fphar.2022.1017129 36210856 PMC9537626

[14] Couto N Monteiro D Cid L Bento T. Effect of different types of exercise in adult subjects with fibromyalgia: a systematic review and meta-analysis of randomized clinical trials. Sci Rep. (2022) 12(1):10391. 10.1038/s41598-022-14213-x 35725780 PMC9209512

[15] Estévez-López F Maestre-Cascales C Russell D Álvarez-Gallardo IC Rodriguez-Ayllon M Hughes CM Effectiveness of exercise on fatigue and sleep quality in fibromyalgia: a systematic review and meta-analysis of randomized trials. Arch Phys Med Rehabil. (2021) 102(4):752–61. 10.1016/j.apmr.2020.06.019 32721388

[16] Häuser W Walitt B Fitzcharles MA Sommer C. Review of pharmacological therapies in fibromyalgia syndrome. Arthritis Res Ther. (2014) 16:201. 10.1186/ar4441 24433463 PMC3979124

[17] Lombardo M Feraco A Ottaviani M Rizzo G Camajani E Caprio M The efficacy of vitamin D supplementation in the treatment of fibromyalgia syndrome and chronic musculoskeletal pain. Nutrients. (2022) 14(15):3010. 10.3390/nu14153010 35893864 PMC9330000

[18] Lowry E Marley J McVeigh JG McSorley E Allsopp P Kerr D. Dietary interventions in the management of fibromyalgia: a systematic review and best-evidence synthesis. Nutrients. (2020) 12(9):2664. 10.3390/nu12092664 32878326 PMC7551150

[19] Maeda T Kudo Y Horiuchi T Makino N. Clinical and anti-aging effect of mud-bathing therapy for patients with fibromyalgia. Mol Cell Biochem. (2018) 444(1–2):87–92. 10.1007/s11010-017-3233-4 29214470

[20] Nadal-Nicolás Y Rubio-Arias JÁ Martínez-Olcina M Reche-García C Hernández-García M Martínez-Rodríguez A. Effects of manual therapy on fatigue, pain, and psychological aspects in women with fibromyalgia. Int J Environ Res Public Health. (2020) 17(12):4611. 10.3390/ijerph17124611 32604939 PMC7345776

[21] Nielsen SS Skou ST Larsen AE Bricca A Søndergaard J Christensen JR. The effect of occupational engagement on lifestyle in adults living with chronic pain: a systematic review and meta-analysis. Occup Ther Int. (2022) 2022:7082159. 10.1155/2022/7082159 35814357 PMC9208937

[22] Vlaeyen JW Teeken-Gruben NJ Goossens ME Rutten-van Mölken MP Pelt RA van Eek H Cognitive-educational treatment of fibromyalgia: a randomized clinical trial. I. Clinical effects. J Rheumatol. (1996) 23(7):1237–45. 8823699

[23] Littlejohn G Guymer E. Neurogenic inflammation in fibromyalgia. Semin Immunopathol. (2018) 40(3):291–300. 10.1007/s00281-018-0672-2 29556959

[24] Staud R Vierck CJ Cannon RL Mauderli AP Price DD. Abnormal sensitization and temporal summation of second pain (wind-up) in patients with fibromyalgia syndrome. Pain. (2001) 91(1–2):165–75. 10.1016/s0304-3959(00)00432-2 11240089

[25] Desmeules JA Cedraschi C Rapiti E Baumgartner E Finckh A Cohen P Neurophysiologic evidence for a central sensitization in patients with fibromyalgia. Arthritis Rheum. (2003) 48(5):1420–9. 10.1002/art.10893 12746916

[26] López-Solà M Woo CW Pujol J Deus J Harrison BJ Monfort J Towards a neurophysiological signature for fibromyalgia. Pain. (2017) 158(1):34–47. 10.1097/j.pain.0000000000000707 27583567 PMC5161739

[27] Feraco P Nigro S Passamonti L Grecucci A Caligiuri ME Gagliardo C Neurochemical correlates of brain atrophy in fibromyalgia syndrome: a magnetic resonance spectroscopy and cortical thickness study. Brain Sci. (2020) 10(6):395. 10.3390/brainsci10060395 32575715 PMC7349375

[28] Napadow V Harris RE. What has functional connectivity and chemical neuroimaging in fibromyalgia taught US about the mechanisms and management of ‘centralized’ pain? Arthritis Res Ther. (2014) 16:1–8. 10.1186/s13075-014-0425-0 PMC4289059 25606591

[29] Pyke TL Osmotherly PG Baines S. Measuring glutamate levels in the brains of fibromyalgia patients and a potential role for glutamate in the pathophysiology of fibromyalgia symptoms: a systematic review. Clin J Pain. (2017) 33(10):944–54. 10.1097/AJP.0000000000000474 28033157

[30] Sawaddiruk P Paiboonworachat S Chattipakorn N Chattipakorn SC. Alterations of brain activity in fibromyalgia patients. J Clin Neurosci. (2017) 38:13–22. 10.1016/j.jocn.2016.12.014 28087191

[31] Zhang Y Lu X Bhavnani BR. Equine estrogens differentially inhibit DNA fragmentation induced by glutamate in neuronal cells by modulation of regulatory proteins involved in programmed cell death. BMC Neurosci. (2003) 4:32. 10.1186/1471-2202-4-32 14693041 PMC340384

[32] Jung YH Kim H Lee D Lee JY Lee WJ Moon JY Abnormal neurometabolites in fibromyalgia patients: magnetic resonance spectroscopy study. Mol Pain. (2021) 17:1744806921990946. 10.1177/1744806921990946 33573464 PMC7887674

[33] Zohuri B McDaniel P. Chapter 6: Electrical brain stimulation to treat neurological disorders. In: Zohuri B McDaniel P, editors. Transcranial Magnetic and Electrical Brain Stimulation for Neurological Disorders. San Diego, CA: Academic Press (2022). p. 267–302.

[34] BrainSTIMCenter. Transcranial electrical stimulation (2024). Available online at: https://www.med.upenn.edu/brainstimcenter/transcranial-electric-stimulation-tes.html (Accessed January 07, 2024).

[35] Moreno-Duarte I Gebodh N Schestatsky P Guleyupoglu B Reato D Bikson M Chapter 2: Transcranial electrical stimulation: transcranial direct current stimulation (tDCS), transcranial alternating current stimulation (tACS), transcranial pulsed current stimulation (tPCS), and transcranial random noise stimulation (tRNS). In: Kadosh RC, editor. The Stimulated Brain. San Diego, CA: Academic Press (2014). p. 35–59.

[36] Starnes K Schulze-Bonhage A Lundstrom B. Chapter 7: Noninvasive brain stimulation for epilepsy. In: Rao VR, editor. Neurostimulation for Epilepsy. San Diego, CA: Academic Press (2023). p. 175–94.

[37] Helfrich RF Schneider TR Rach S Trautmann-Lengsfeld SA Engel AK Herrmann CS. Entrainment of brain oscillations by transcranial alternating current stimulation. Curr Biol. (2014) 24(3):333–9. 10.1016/j.cub.2013.12.041 24461998

[38] Thair H Holloway AL Newport R Smith AD. Transcranial direct current stimulation (tDCS): a beginner’s guide for design and implementation. Front Neurosci. (2017) 11:641. 10.3389/fnins.2017.00641 29213226 PMC5702643

[39] Garnett EO Malyutina S Datta A den Ouden D-B. On the use of the terms anodal and cathodal in high-definition transcranial direct current stimulation: a technical note. Neuromodulation. (2015) 18(8):705–13. 10.1111/ner.12320 26076228

[40] Korai SA Ranieri F Di Lazzaro V Papa M Cirillo G. Neurobiological after-effects of low intensity transcranial electric stimulation of the human nervous system: from basic mechanisms to metaplasticity. Front Neurol. (2021) 12:587771. 10.3389/fneur.2021.587771 33658972 PMC7917202

[41] Antal A Paulus W. Transcranial alternating current stimulation (tACS). Front Hum Neurosci. (2013) 7:317. 10.3389/fnhum.2013.00317 23825454 PMC3695369

[42] Hopfinger JB Parsons J Fröhlich F. Differential effects of 10-hz and 40-hz transcranial alternating current stimulation (tACS) on endogenous versus exogenous attention. Cogn Neurosci. (2017) 8(2):102–11. 10.1080/17588928.2016.1194261 27297977

[43] Nomura T Asao A Kumasaka A. Transcranial alternating current stimulation over the prefrontal cortex enhances episodic memory recognition. Exp Brain Res. (2019) 237(7):1709–15. 10.1007/s00221-019-05543-w 31011766

[44] Santarnecchi E Muller T Rossi S Sarkar A Polizzotto NR Rossi A Individual differences and specificity of prefrontal gamma frequency-tACS on fluid intelligence capabilities. Cortex. (2016) 75:33–43. 10.1016/j.cortex.2015.11.003 26707084

[45] De Ridder D Stöckl T To WT Langguth B Vanneste S. Chapter 7: Noninvasive transcranial magnetic and electrical stimulation: working mechanisms. In: Evans JR Turner RP, editors. Rhythmic Stimulation Procedures in Neuromodulation. San Diego, CA: Academic Press (2017). p. 193–223.

[46] Motolese F Capone F Di Lazzaro V. Chapter 21: New tools for shaping plasticity to enhance recovery after stroke. In: Quartarone A Ghilardi MF Boller F, editors. Handbook of Clinical Neurology, Vol. 184. Amsterdam: Elsevier (2022). p. 299–315. 10.1016/B978-0-12-819410-2.00016-3 35034743

[47] Inukai Y Saito K Sasaki R Tsuiki S Miyaguchi S Kojima S Comparison of three non-invasive transcranial electrical stimulation methods for increasing cortical excitability. Front Hum Neurosci. (2016) 10:668. 10.3389/fnhum.2016.00668 28082887 PMC5186778

[48] Murphy OW Hoy KE Wong D Bailey NW Fitzgerald PB Segrave RA. Transcranial random noise stimulation is more effective than transcranial direct current stimulation for enhancing working memory in healthy individuals: behavioural and electrophysiological evidence. Brain Stimul. (2020) 13(5):1370–80. 10.1016/j.brs.2020.07.001 32659482

[49] Curatolo M La Bianca G Cosentino G Baschi R Salemi G Talotta R Motor cortex tRNS improves pain, affective and cognitive impairment in patients with fibromyalgia: preliminary results of a randomised sham-controlled trial. Clin Exp Rheumatol. (2017) 35(Suppl 105):100–5. 28681715

[50] Palm U Chalah MA Padberg F Al-Ani T Abdellaoui M Sorel M Effects of transcranial random noise stimulation (tRNS) on affect, pain and attention in multiple sclerosis. Restor Neurol Neurosci. (2016) 34(2):189–99. 10.3233/RNN-150557 26890095

[51] Clauw DJ Arnold LM McCarberg BH, FibroCollaborative. The science of fibromyalgia. Mayo Clin Proc. (2011) 86(9):907–11. 10.4065/mcp.2011.0206 21878603 PMC3258006

[52] Kim J Loggia ML Cahalan CM Harris RE Beissner F Garcia RG The somatosensory link in fibromyalgia: functional connectivity of the primary somatosensory cortex is altered by sustained pain and is associated with clinical/autonomic dysfunction. Arthritis Rheumatol. (2015) 67(5):1395–405. 10.1002/art.39043 25622796 PMC4414820

[53] Teng H-W Tani J Chang T-S Chen H-J Lin Y-C Lin CS-Y Altered sensory nerve excitability in fibromyalgia. J Formos Med Assoc. (2021) 120(8):1611–9. 10.1016/j.jfma.2021.02.003 33642123

[54] Wang S Du S-H Wang X-Q Lu J-Y. Mechanisms of transcranial direct current stimulation (tDCS) for pain in patients with fibromyalgia syndrome. Front Mol Neurosci. (2024) 17:1269636. 10.3389/fnmol.2024.1269636 38356687 PMC10865494

[55] Kang JH Choi SE Park DJ Xu H Lee JK Lee SS. Effects of add-on transcranial direct current stimulation on pain in Korean patients with fibromyalgia. Sci Rep. (2020) 10(1):12114. 10.1038/s41598-020-69131-7 32694653 PMC7374102

[56] Marlow NM Bonilha HS Short EB. Efficacy of transcranial direct current stimulation and repetitive transcranial magnetic stimulation for treating fibromyalgia syndrome: a systematic review. Pain Pract. (2013) 13(2):131–45. 10.1111/j.1533-2500.2012.00562.x 22631436

[57] Azarkolah A Noorbala AA Ansari S Hallajian A-H Salehinejad MA. Efficacy of transcranial direct current stimulation on pain level and disability of patients with fibromyalgia: a systematic review of randomized controlled trials with parallel-group design. Brain Sci. (2024) 14:26. 10.3390/brainsci14010026 PMC10813480 38248241

[58] Cheng YC Hsiao CY Su MI Chiu CC Huang YC Huang WL. Treating fibromyalgia with electrical neuromodulation: a systematic review and meta-analysis. Clin Neurophysiol. (2023) 148:17–28. 10.1016/j.clinph.2023.01.011 36774784

[59] Lloyd DM Wittkopf PG Arendsen LJ Jones AKP. Is transcranial direct current stimulation (tDCS) effective for the treatment of pain in fibromyalgia? A systematic review and meta-analysis. J Pain. (2020) 21(11–12):1085–100. 10.1016/j.jpain.2020.01.003 31982685

[60] Teixeira PEP Pacheco-Barrios K Branco LC de Melo PS Marduy A Caumo W The analgesic effect of transcranial direct current stimulation in fibromyalgia: a systematic review, meta-analysis, and meta-regression of potential influencers of clinical effect. Neuromodulation. (2023) 26(4):715–27. 10.1016/j.neurom.2022.10.044 36435660 PMC10203058

[61] Yang CL Qu Y Huang JP Wang TT Zhang H Chen Y Efficacy and safety of transcranial direct current stimulation in the treatment of fibromyalgia: a systematic review and meta-analysis. Neurophysiol Clin. (2024) 54(1):102944. 10.1016/j.neucli.2024.102944 38387108

[62] Schardt C Adams MB Owens T Keitz S Fontelo P. Utilization of the PICO framework to improve searching PubMed for clinical questions. BMC Med Inform Decis Mak. (2007) 7:16. 10.1186/1472-6947-7-16 17573961 PMC1904193

[63] Page MJ McKenzie JE Bossuyt PM Boutron I Hoffmann TC Mulrow CD The PRISMA 2020 statement: an updated guideline for reporting systematic reviews. Br Med J. (2021) 372:n71. 10.1136/bmj.n71 33782057 PMC8005924

[64] Higgins JPT Thomas J Chandler J Cumpston M Li T Page MJ, editors. Cochrane Handbook for Systematic Reviews of Interventions version 6.5. London: Cochrane (2024). Available online at: www.cochrane.org/handbook

[65] Kamper SJ Moseley AM Herbert RD Maher CG Elkins MR Sherrington C. 15 Years of tracking physiotherapy evidence on PEDro, where are we now? Br J Sports Med. (2015) 49(14):907–9. 10.1136/bjsports-2014-094468 25833902

[66] Clausen K. Developing an objective measure of pain. Himmelfarb Health Sciences Library Newsletter, the George Washington University School of Medicine and Health Sciences. Health Sciences Research Commons (2023). Available online at: https://healthsciencesresearchcommons.gwu.edu (Accessed January 16, 2025).

[67] Uddin Z MacDermid JC. Quantitative sensory testing in chronic musculoskeletal pain. Pain Med. (2016) 17(9):1694–703. 10.1093/pm/pnv105 26893116

[68] Weaver KR Griffioen MA Klinedinst NJ Galik E Duarte AC Colloca L Quantitative sensory testing across chronic pain conditions and use in special populations. Front Pain Res. (2022) 2:779068. 10.3389/fpain.2021.779068 PMC8915716 35295425

[69] Shafshak TS Elnemr R. The visual analogue scale versus numerical rating scale in measuring pain severity and predicting disability in low back pain. J Clin Rheumatol. (2021) 27(7):282–5. 10.1097/RHU.0000000000001320 31985722

[70] Thong I Jensen M Miró J Tan G. The validity of pain intensity measures: what do the NRS, VAS, VRS, and FPS-R measure? Scand J Pain. (2018) 18(1):99–107. 10.1515/sjpain-2018-0012 29794282

[71] Ye W Hackett S Vandevelde C Twigg S Helliwell PS Coates LC. Comparing the visual analogue scale (VAS) and the numerical rating scale (NRS) in patient reported outcomes in psoriatic arthritis. J Rheumatol. (2020) 48(6):836–40. 10.3899/jrheum.200928 33262305

[72] Pavlaković G Petzke F. The role of quantitative sensory testing in the evaluation of musculoskeletal pain conditions. Curr Rheumatol Rep. (2010) 12:455–61. 10.1007/s11926-010-0131-0 20857243 PMC3128735

[73] van Driel MEC Huygen FJPM Rijsdijk M. Quantitative sensory testing: a practical guide and clinical applications. BJA Educ. (2024) 24(9):326–34. 10.1016/j.bjae.2024.05.004 39234156 PMC11368601

[74] Pace AK Bruceta M Donovan J Vaida SJ Eckert JM. An objective pain score for chronic pain clinic patients. Pain Res Manag. (2021) 2021:6695741. 10.1155/2021/6695741 33628355 PMC7884155

[75] Wideman TH Edwards RR Walton DM Martel MO Hudon A Seminowicz DA. The multimodal assessment model of pain: a novel framework for further integrating the subjective pain experience within research and practice. Clin J Pain. (2019) 35(3):212–21. 10.1097/AJP.0000000000000670 30444733 PMC6382036

[76] Mateen FJ Oh J Tergas AI Bhayani NH Kamdar BB. Titles versus titles and abstracts for initial screening of articles for systematic reviews. Clin Epidemiol. (2013) 5:89–95. 10.2147/CLEP.S43118 23526335 PMC3604876

[77] Huang X Lin J Demner-Fushman D. Evaluation of PICO as a knowledge representation for clinical questions. AMIA Annu Symp Proc. (2006) 2006:359–63. 17238363 PMC1839740

[78] Miller SA Forrest JL. Enhancing your practice through evidence-based decision making: PICO, learning how to ask good questions. J Evid Based Dent Pract. (2001) 1(2):136–41. 10.1016/s1532-3382(01)70024-3

[79] Richardson WS Wilson MC Nishikawa J Hayward RS. The well-built clinical question: a key to evidence-based decisions. ACP J Club. (1995) 123(3):A12–3. 10.7326/ACPJC-1995-123-3-A12 7582737

[80] Wolfe F Clauw DJ Fitzcharles MA Goldenberg DL Katz RS Mease P The American college of rheumatology preliminary diagnostic criteria for fibromyalgia and measurement of symptom severity. Arthritis Care Res. (2010) 62(5):600–10. 10.1002/acr.20140 20461783

[81] Higgins JPT. Rob 2: a revised tool for assessing risk of bias in randomised trials. Br Med J. (2019) 366:l4898. 10.1136/bmj.l4898 31462531

[82] Matos AP Pegorari MS. How to classify clinical trials using the PEDro scale? J Lasers Med Sci. (2020) 11(1):1–2. 10.15171/jlms.2020.01 32099619 PMC7008740

[83] Reichow B Barton EE Maggin DM. Development and applications of the single-case design risk of bias tool for evaluating single-case design research study reports. Res Dev Disabil. (2018) 79:53–64. 10.1016/j.ridd.2018.05.008 29958733

[84] DalĺAgnol L Pascoal-Faria P Barros Cecílio S Corrêa FI. Transcranial direct current stimulation in the neuromodulation of pain in fibromyalgia: a case study. Ann Phys Rehabil Med. (2015) 58(6):351–3. 10.1016/j.rehab.2015.10.002 26589350

[85] Higgins JP Altman DG Gøtzsche PC Jüni P Moher D Oxman AD The cochrane collaboration’s tool for assessing risk of bias in randomised trials. Br Med J. (2011) 343:d5928. 10.1136/bmj.d5928 22008217 PMC3196245

[86] Sterne JAC Savović J Page MJ Elbers RG Blencowe NS Boutron I Rob 2: a revised tool for assessing risk of bias in randomized trials. Br Med J. (2019) 366:l4898. 10.1136/bmj.l4898 31462531

[87] Ding H Hu GL Zheng XY Chen Q Threapleton DE Zhou ZH. The method quality of cross-over studies involved in cochrane systematic reviews. PLoS One. (2015) 10(4):e0120519. 10.1371/journal.pone.0120519 25867772 PMC4395015

[88] Landis JR Koch GG. The measurement of observer agreement for categorical data. Biometrics. (1977) 33(1):159–74. 10.2307/2529310 843571

[89] Arroyo-Fernández R Avendaño-Coy J Velasco-Velasco R Palomo-Carrión R Bravo-Esteban E Ferri-Morales A. Effectiveness of transcranial direct current stimulation combined with exercising in people with fibromyalgia: a randomized sham-controlled clinical trial. Arch Phys Med Rehabil. (2022) 103(8):1524–32. 10.1016/j.apmr.2022.02.020 35331718

[90] Velickovic Z Radunovic G. Repetitive transcranial magnetic stimulation in fibromyalgia: exploring the necessity of neuronavigation for targeting new brain regions. J Pers Med. (2024) 14:662. 10.3390/jpm14060662 38929883 PMC11204413

[91] Tiwari VK Kumar A Nanda S Chaudhary S Sharma R Kumar U Effect of neuronavigated repetitive transcranial magnetic stimulation on pain, cognition and cortical excitability in fibromyalgia syndrome. Neurol Sci. (2024) 45:3421–33. 10.1007/s10072-024-07343-9 38270728

[92] Brietzke AP Zortea M Carvalho F Sanches PRS Silva DPJ Torres ILDS Large treatment effect with extended home-based transcranial direct current stimulation over dorsolateral prefrontal cortex in fibromyalgia: a proof of concept sham-randomized clinical study. J Pain. (2020) 21(1–2):212–24. 10.1016/j.jpain.2019.06.013 31356985

[93] Khedr EM Omran EAH Ismail NM El-Hammady DH Goma SH Kotb H Effects of transcranial direct current stimulation on pain, mood and serum endorphin level in the treatment of fibromyalgia: a double blinded, randomized clinical trial. Brain Stimul. (2017) 10(5):893–901. 10.1016/j.brs.2017.06.006 28684258

[94] Lim M Kim DJ Nascimento TD Ichesco E Kaplan C Harris RE Functional magnetic resonance imaging signal variability is associated with neuromodulation in fibromyalgia. Neuromodulation. (2023) 26(5):999–1008. 10.1111/ner.13512 34309138 PMC8789944

[95] Loreti EH Freire AM Alexandre da Silva A Kakuta E Martins Neto UR Konkiewitz EC. Effects of anodal transcranial direct current stimulation on the primary motor cortex in women with fibromyalgia: a randomized, triple-blind clinical trial. Neuromodulation. (2023) 26(4):767–77. 10.1016/j.neurom.2022.11.007 36702675

[96] Monte-Silva K Kuo MF Liebetanz D Paulus W Nitsche MA. Shaping the optimal repetition interval for cathodal transcranial direct current stimulation (tDCS). J Neurophysiol. (2010) 103(4):1735–40. 10.1152/jn.00924.2009 20107115

[97] Fregni F El-Hagrassy MM Pacheco-Barrios K Carvalho S Leite J Simis M Evidence-based guidelines and secondary meta-analysis for the use of transcranial direct current stimulation in neurological and psychiatric disorders. Int J Neuropsychopharmacol. (2021) 24(4):256–313. 10.1093/ijnp/pyaa051 32710772 PMC8059493

[98] Mendonca ME Simis M Grecco LC Battistella LR Baptista AF Fregni F. Transcranial direct current stimulation combined with aerobic exercise to optimize analgesic responses in fibromyalgia: a randomized placebo-controlled clinical trial. Front Hum Neurosci. (2016) 10:68. 10.3389/fnhum.2016.00068 27014012 PMC4785149

[99] Matias MGL Germano Maciel D França IM Cerqueira MS Silva TCLA Okano AH Transcranial direct current stimulation associated with functional exercise program for treating fibromyalgia: a randomized controlled trial. Arch Phys Med Rehabil. (2022) 103(2):245–54. 10.1016/j.apmr.2021.06.029 34480887

[100] Ramasawmy P Khalid S Petzke F Antal A. Pain reduction in fibromyalgia syndrome through pairing transcranial direct current stimulation and mindfulness meditation: a randomized, double-blinded, sham-controlled pilot clinical trial. Front Med. (2022) 9:908133. 10.3389/fmed.2022.908133 PMC9596988 36314032

[101] Yoo HB Ost J Joos W Van Havenbergh T De Ridder D Vanneste S. Adding prefrontal transcranial direct current stimulation before occipital nerve stimulation in fibromyalgia. Clin J Pain. (2018) 34(5):421–7. 10.1097/AJP.0000000000000552 28877142

[102] Samartin-Veiga N González-Villar AJ Pidal-Miranda M Vázquez-Millán A Carrillo-de-la-Peña MT. Active and sham transcranial direct current stimulation (tDCS) improved quality of life in female patients with fibromyalgia. Qual Life Res. (2022) 31(8):2519–34. 10.1007/s11136-022-03106-1 35229253 PMC9250466

[103] Villamar MF Wivatvongvana P Patumanond J Bikson M Truong DQ Datta A Focal modulation of the primary motor cortex in fibromyalgia using 4×1-ring high-definition transcranial direct current stimulation (HD-tDCS): immediate and delayed analgesic effects of cathodal and anodal stimulation. J Pain. (2013) 14(4):371–83. 10.1016/j.jpain.2012.12.007 23415877

[104] Forogh B Haqiqatshenas H Ahadi T Ebadi S Alishahi V Sajadi S. Repetitive transcranial magnetic stimulation (rTMS) versus transcranial direct current stimulation (tDCS) in the management of patients with fibromyalgia: a randomized controlled trial. Neurophysiol Clin. (2021) 51(4):339–47. 10.1016/j.neucli.2021.03.002 33814258

[105] Serrano PV Zortea M Alves RL Beltrán G Bavaresco C Ramalho L The effect of home-based transcranial direct current stimulation in cognitive performance in fibromyalgia: a randomized, double-blind sham-controlled trial. Front Hum Neurosci. (2022) 16:992742. 10.3389/fnhum.2022.992742 36504629 PMC9730884

[106] Samartin-Veiga N Pidal-Miranda M González-Villar AJ Bradley C Garcia-Larrea L O'Brien AT Transcranial direct current stimulation of 3 cortical targets is no more effective than placebo as treatment for fibromyalgia: a double-blind sham-controlled clinical trial. Pain. (2022) 163(7):e850–61. 10.1097/j.pain.0000000000002493 34561393

[107] Caumo W Alves RL Vicuña P Alves CFDS Ramalho L Sanches PRS Impact of bifrontal home-based transcranial direct current stimulation in pain catastrophizing and disability due to pain in fibromyalgia: a randomized, double-blind sham-controlled study. J Pain. (2022) 23(4):641–56. 10.1016/j.jpain.2021.11.002 34785366

[108] Santos VSDSD Zortea M Alves RL Naziazeno CCDS Saldanha JS Carvalho SDCR Cognitive effects of transcranial direct current stimulation combined with working memory training in fibromyalgia: a randomized clinical trial. Sci Rep. (2018) 8(1):12477. 10.1038/s41598-018-30127-z 30127510 PMC6102237

[109] Foerster BR Nascimento TD DeBoer M Bender MA Rice IC Truong DQ Excitatory and inhibitory brain metabolites as targets of motor cortex transcranial direct current stimulation therapy and predictors of its efficacy in fibromyalgia. Arthritis Rheumatol. (2015) 67(2):576–81. 10.1002/art.38945 25371383 PMC4446981

[110] Cummiford CM Nascimento TD Foerster BR Clauw DJ Zubieta JK Harris RE Changes in resting state functional connectivity after repetitive transcranial direct current stimulation applied to motor cortex in fibromyalgia patients. Arthritis Res Ther. (2016) 18:40. 10.1186/s13075-016-0934-0 26842987 PMC4741001

[111] de Melo GA de Oliveira EA Dos Santos Andrade SMM Fernández-Calvo B Torro N. Comparison of two tDCS protocols on pain and EEG alpha-2 oscillations in women with fibromyalgia. Sci Rep. (2020) 10(1):18955. 10.1038/s41598-020-75861-5 33144646 PMC7609530

[112] Schein B Beltran G França BR Sanches PRS Silva DP Jr Torres IL Effects of hypnotic analgesia and transcranial direct current stimulation on pain tolerance and corticospinal excitability in individuals with fibromyalgia: a cross-over randomized clinical trial. J Pain Res. (2023) 16:187–203. 10.2147/JPR.S384373 36718400 PMC9884000

[113] Rocca M Clemente L Gentile E Ricci K Delussi M de Tommaso M. Effect of single session of anodal M1 transcranial direct current stimulation-TDCS-on cortical hemodynamic activity: a pilot study in fibromyalgia. Brain Sci. (2022) 12(11):1569. 10.3390/brainsci12111569 36421893 PMC9688269

[114] Silva AF Zortea M Carvalho S Leite J Torres IL Fregni F Anodal transcranial direct current stimulation over the left dorsolateral prefrontal cortex modulates attention and pain in fibromyalgia: randomized clinical trial. Sci Rep. (2017) 7(1):135. 10.1038/s41598-017-00185-w 28273933 PMC5427889

[115] Paula TMH Castro MS Medeiros LF Paludo RH Couto FF Costa TRD Association of low-dose naltrexone and transcranial direct current stimulation in fibromyalgia: a randomized, double-blinded, parallel clinical trial. Braz J Anesthesiol. (2023) 73(4):409–17. 10.1016/j.bjane.2022.08.003 35988815 PMC10362456

[116] Castillo-Saavedra L Gebodh N Bikson M Diaz-Cruz C Brandao R Coutinho L Clinically effective treatment of fibromyalgia pain with high-definition transcranial direct current stimulation: phase II open-label dose optimization. J Pain. (2016) 17(1):14–26. 10.1016/j.jpain.2015.09.009 26456677 PMC5777157

[117] Fagerlund AJ Hansen OA Aslaksen PM. Transcranial direct current stimulation as a treatment for patients with fibromyalgia: a randomized controlled trial. Pain. (2015) 156(1):62–71. 10.1016/j.pain.0000000000000006 25599302

[118] Jales LH Jr Costa MD Jales LH Ribeiro JP Freitas WJ Teixeira MJ. Transcranial direct current stimulation in fibromyalgia: effects on pain and quality of life evaluated clinically and by brain perfusion scintigraphy. Revista Dor. (2015) 16(1):37–42. 10.5935/1806-0013.20150008

[119] Matias MGL Cavalcante AFL Mescouto KA Silva Filho EM Baptista AF Okano AH Does anodal transcranial direct current stimulation over left motor cortex show body side pain-related difference in fibromyalgia? Braz J Pain. (2022) 5(2):112–8. 10.5935/2595-0118.20220020-pt

[120] To WT James E Ost J Hart J Jr De Ridder D Vanneste S. Differential effects of bifrontal and occipital nerve stimulation on pain and fatigue using transcranial direct current stimulation in fibromyalgia patients. J Neural Transm. (2017) 124(7):799–808. 10.1007/s00702-017-1714-y 28321566

[121] Behnoush AH Khalaji A Khanmohammadi S Alehossein BS Shobeiri P Teixeira AL Brain-derived neurotrophic factor in fibromyalgia: a systematic review and meta-analysis of its role as a potential biomarker. PLoS One. (2023) 18:e0296103. 10.1371/journal.pone.0296103 38127937 PMC10734974

[122] Miranda M Morici JF Zanoni MB Bekinschtein P. Brain-derived neurotrophic factor: a key molecule for memory in the healthy and the pathological brain. Front Cell Neurosci. (2019) 13:363. 10.3389/fncel.2019.00363 31440144 PMC6692714

[123] Yamada K Mizuno M Nabeshima T. Role for brain-derived neurotrophic factor in learning and memory. Life Sci. (2002) 70(7):735–44. 10.1016/s0024-3205(01)01461-8 11833737

[124] Naegelin Y Dingsdale H Säuberli K Schädelin S Kappos L Barde YA. Measuring and validating the levels of brain-derived neurotrophic factor in human serum. eNeuro. (2018) 5(2):ENEURO.0419-17.2018. 10.1523/ENEURO.0419-17.2018 29662942 PMC5898630

[125] Navarro López J Moral Bergós RD Marijuán PC. Significant new quantitative EEG patterns in fibromyalgia. Eur J Psychiatry. (2015) 29(4):277–92. 10.4321/S0213-61632015000400005

[126] Villafaina S Collado-Mateo D Fuentes-García JP Cano-Plasencia R Gusi N. Impact of fibromyalgia on alpha-2 EEG power spectrum in the resting condition: a descriptive correlational study. BioMed Res Int. (2019) 2019:7851047. 10.1155/2019/7851047 31058192 PMC6463609

[127] Sterling P. Allostasis: a model of predictive regulation. Physiol Behav. (2012) 106(1):5–15. 10.1016/j.physbeh.2011.06.004 21684297

[128] Vanneste S Ost J Van Havenbergh T De Ridder D. Resting state electrical brain activity and connectivity in fibromyalgia. PLoS One. (2017) 12(6):e0178516. 10.1371/journal.pone.0178516 28650974 PMC5484465

[129] Fischer AA. Pressure threshold meter: its use for quantification of tender spots. Arch Phys Med Rehabil. (1986) 67(11):836–8. 10.1016/0304-3959(87)90089-3 3778185

[130] Freitas RP Andrade SC Carvalho RF Sousa MB. Pressure pain endurance in women with fibromyalgia. Revista Dor. (2014) 15(4):260–3. 10.5935/1806-0013.20140056

[131] Maquet D Croisier JL Demoulin C Crielaard JM. Pressure pain thresholds of tender point sites in patients with fibromyalgia and in healthy controls. Eur J Pain. (2004) 8(2):111–7. 10.1016/S1090-3801(03)00082-X 14987620

[132] Mikkelsson M Latikka P Kautiainen H Isomeri R Isomäki H. Muscle and bone pressure pain threshold and pain tolerance in fibromyalgia patients and controls. Arch Phys Med Rehabil. (1992) 73(9):814–8. 10.5555/uri:pii:000399939290151l 1514890

[133] Navarro-Ledesma S Aguilar-García M González-Muñoz A Casas-Barragán A Tapia-Haro RM. Association between elasticity of tissue and pain pressure threshold in the tender points present in subjects with fibromyalgia: a cross-sectional study. Sci Rep. (2023) 13:22003. 10.1038/s41598-023-49550-y 38086996 PMC10716166

[134] Pilozzi A Carro C Huang X. Roles of β-endorphin in stress, behavior, neuroinflammation, and brain energy metabolism. Int J Mol Sci. (2020) 22(1):338. 10.3390/ijms22010338 33396962 PMC7796446

[135] Bidari A Ghavidel-Parsa B Rajabi S Sanaei O Toutounchi M. The acute effect of maximal exercise on plasma beta-endorphin levels in fibromyalgia patients. Korean J Pain. (2016) 29(4):249–54. 10.3344/kjp.2016.29.4.249 27738503 PMC5061641

[136] Panerai AE Vecchiet J Panzeri P Meroni P Scarone S Pizzigallo E Peripheral blood mononuclear cell beta-endorphin concentration is decreased in chronic fatigue syndrome and fibromyalgia but not in depression: preliminary report. Clin J Pain. (2002) 18(4):270–3. 10.1097/00002508-200207000-00008 12131069

[137] Schmidt-Wilcke T Clauw DJ. Fibromyalgia: from pathophysiology to therapy. Nat Rev Rheumatol. (2011) 7(9):518–27. 10.1038/nrrheum.2011.98 21769128

[138] Mhalla A de Andrade DC Baudic S Perrot S Bouhassira D. Alteration of cortical excitability in patients with fibromyalgia. Pain. (2010) 149(3):495–500. 10.1016/j.pain.2010.03.009 20356675

[139] Pacheco-Barrios K Lima D Pimenta D Slawka E Navarro-Flores A Parente J Motor cortex inhibition as a fibromyalgia biomarker: a meta-analysis of transcranial magnetic stimulation studies. Brain Netw Modul. (2022) 1(2):88–101. 10.4103/2773-2398.348254 35845034 PMC9282159

[140] Castelo-Branco L Cardenas-Rojas A Pacheco-Barrios K Teixeira PEP Gonzalez-Mego P Vasquez-Avila K Can neural markers be used for fibromyalgia clinical management? Princ Pract Clin Res. (2022) 8(1):28–33. 10.21801/ppcrj.2022.81.5 35677778 PMC9172964

[141] Passard A Attal N Benadhira R Brasseur L Saba G Sichere P Effects of unilateral repetitive transcranial magnetic stimulation of the motor cortex on chronic widespread pain in fibromyalgia. Brain J Neurol. (2007) 130(Pt 10):2661–70. 10.1093/brain/awm189 17872930

[142] Rossini PM Burke D Chen R Cohen LG Daskalakis Z Di Iorio R Non-invasive electrical and magnetic stimulation of the brain, spinal cord, roots and peripheral nerves: basic principles and procedures for routine clinical and research application. An updated report from an I.F.C.N. committee. Clin Neurophysiol. (2015) 126(6):1071–107. 10.1016/j.clinph.2015.02.001 25797650 PMC6350257

[143] Tremblay S Beaulé V Proulx S de Beaumont L Marjanska M Doyon J Relationship between transcranial magnetic stimulation measures of intracortical inhibition and spectroscopy measures of GABA and glutamate+glutamine. J Neurophysiol. (2013) 109(5):1343–9. 10.1152/jn.00704.2012 23221412 PMC3602833

[144] Zeugin D Ionta S. Anatomo-functional origins of the cortical silent period: spotlight on the basal ganglia. Brain Sci. (2021) 11(6):705. 10.3390/brainsci11060705 34071742 PMC8227635

[145] Kravitz HM Katz RS. Fibrofog and fibromyalgia: a narrative review and implications for clinical practice. Rheumatol Int. (2015) 35(7):1115–25. 10.1007/s00296-014-3208-7 25583051

[146] Muñoz Ladrón de Guevara C Fernández-Serrano MJ Reyes Del Paso GA Duschek S. Executive function impairments in fibromyalgia syndrome: relevance of clinical variables and body mass index. PLoS One. (2018) 13(4):e0196329. 10.1371/journal.pone.0196329 29694417 PMC5918817

[147] Wu YL Huang CJ Fang SC Ko LH Tsai PS. Cognitive impairment in fibromyalgia: a meta-analysis of case-control studies. Psychosom Med. (2018) 80(5):432–8. 10.1097/PSY.0000000000000575 29528888

[148] Ambrosini A De Pasqua V Áfra J Sandor PS Schoenen J. Reduced gating of middle-latency auditory evoked potentials (P50) in migraine patients: another indication of abnormal sensory processing? Neurosci Lett. (2001) 306(1–2):132–4. 10.1016/S0304-3940(01)01871-7 11403975

[149] Montoya P Sitges C García-Herrera M Rodríguez-Cotes A Izquierdo R Truyols M Reduced brain habituation to somatosensory stimulation in patients with fibromyalgia. Arthritis Rheum. (2006) 54(6):1995–2003. 10.1002/art.21910 16732548

[150] Montoro CI Duschek S Schuepbach D Gandarillas M Reyes del Paso GA. Cerebral blood flow variability in fibromyalgia syndrome: relationships with emotional, clinical and functional variables. PLoS One. (2018) 13(9):e0204267. 10.1371/journal.pone.0204267 30235315 PMC6147545

[151] PEDro scale (1999). Available online at: http://www.pedro.org.au/wp-content/uploads/PEDro_scale.pdf (Accessed June 05, 2023).

[152] Elkins MR Moseley AM. Intention-to-treat analysis. J Physiother. (2015) 61(3):165–7. 10.1016/j.jphys.2015.05.013 26096012

[153] Slim K Nini E Forestier D Kwiatkowski F Panis Y Chipponi J. Methodological index for non-randomized studies (minors): development and validation of a new instrument. ANZ J Surg. (2003) 73(9):712–6. 10.1046/j.1445-2197.2003.02748.x 12956787

[154] Alsinbili A. Assessing the risk of bias in single-arm trials for systematic reviews: moving towards a more reliable evaluation of clinical evidence. Future Healthc J. (2023) 10(Suppl 3):26–7. 10.7861/fhj.10-3-s26 38406726 PMC10884709

[155] Cashin AG McAuley JH. Clinimetrics: physiotherapy evidence database (PEDro) scale. J Physiother. (2020) 66(1):59. 10.1016/j.jphys.2019.08.005 31521549

[156] Jo NG Kim GW Won YH Park SH Seo JH Ko MH. Timing-dependent effects of transcranial direct current stimulation on hand motor function in healthy individuals: a randomized controlled study. Brain Sci. (2021) 11(10):1325. 10.3390/brainsci11101325 34679390 PMC8534210

[157] Liao WW Chiang WC Lin KC Wu CY Liu CT Hsieh YW Timing-dependent effects of transcranial direct current stimulation with mirror therapy on daily function and motor control in chronic stroke: a randomized controlled pilot study. J Neuroeng Rehabil. (2020) 17(1):101. 10.1186/s12984-020-00722-1 32690032 PMC7370428

[158] Wen H-Z Gao S-H Zhao Y-D He W-J Tian X-L Ruan H-Z. Parameter optimization analysis of prolonged analgesia effect of tDCS on neuropathic pain rats. Front Behav Neurosci. (2017) 11:115. 10.3389/fnbeh.2017.00115 28659772 PMC5468406

[159] Willigenburg NW Poolman RW. The difference between statistical significance and clinical relevance. The case of minimal important change, non-inferiority trials, and smallest worthwhile effect. Injury. (2023) 54(Suppl 5):110764. 10.1016/j.injury.2023.04.051 37923502

[160] Copay AG Subach BR Glassman SD Polly DW Jr Schuler TC. Understanding the minimum clinically important difference: a review of concepts and methods. Spine J. (2007) 7(5):541–6. 10.1016/j.spinee.2007.01.008 17448732

[161] Bennett RM Bushmakin AG Cappelleri JC Zlateva G Sadosky AB. Minimal clinically important difference in the fibromyalgia impact questionnaire. J Rheumatol. (2009) 36(6):1304–11. 10.3899/jrheum.081090 19369473

[162] Mease PJ Spaeth M Clauw DJ Arnold LM Bradley LA Russell IJ Estimation of minimum clinically important difference for pain in fibromyalgia†. Arthritis Care Res. (2011) 63:821–6. 10.1002/acr.20449 21312349

[163] Farrar JT Young JP Jr LaMoreaux L Werth JL Poole MR. Clinical importance of changes in chronic pain intensity measured on an 11-point numerical pain rating scale. Pain. (2001) 94(2):149–58. 10.1016/S0304-3959(01)00349-9 11690728

[164] Olsen MF Bjerre E Hansen MD Tendal B Hilden J Hróbjartsson A. Minimum clinically important differences in chronic pain vary considerably by baseline pain and methodological factors: systematic review of empirical studies. J Clin Epidemiol. (2018) 101:87–106.e2. 10.1016/j.jclinepi.2018.05.007 29793007

[165] Straudi S Antonioni A Baroni A Bonsangue V Lavezzi S Koch G Anti-inflammatory and cortical responses after transcranial direct current stimulation in disorders of consciousness: an exploratory study. J Clin Med. (2024) 13(1):108. 10.3390/jcm13010108 PMC10779892 38202115

[166] Karadağ A Hayta E Çelik VK Bakir S. Serum vascular endothelial growth factor and vascular endothelial growth factor receptor-1 levels in patients with fibromyalgia syndrome. Arch Rheumatol. (2019) 34(4):414–8. 10.5606/ArchRheumatol.2019.7265 32010890 PMC6974391

[167] Tapia-Haro RM Molina F Rus A Casas-Barragán A Correa-Rodríguez M Aguilar-Ferrándiz ME. Serum VEGF and CGRP biomarkers: relationships with pain intensity, electric pain, pressure pain threshold, and clinical symptoms in fibromyalgia—an observational study. Int J Mol Sci. (2023) 24(21):15533. 10.3390/ijms242115533 37958517 PMC10649295

[168] Findeisen K Guymer E Littlejohn G. Neuroinflammatory and immunological aspects of fibromyalgia. Brain Sci. (2025) 15(2):206. 10.3390/brainsci15020206 40002538 PMC11852494

[169] Yao M Wang S Han Y Zhao H Yin Y Zhang Y Micro-inflammation related gene signatures are associated with clinical features and immune status of fibromyalgia. J Transl Med. (2023) 21:77. 10.1186/s12967-023-04477-w 37670381 PMC10478377

[170] Bains A Kohrman S Punko D Fricchione G. A link between inflammatory mechanisms and fibromyalgia. In: Kim YK, editor. Neuroinflammation, Gut-Brain Axis and Immunity in Neuropsychiatric Disorders. Advances in Experimental Medicine and Biology. Vol 1411. Singapore: Springer (2023). 10.1007/978-981-19-7376-5_16 36949318

[171] Seminowicz DA Moayedi M. The dorsolateral prefrontal cortex in acute and chronic pain. J Pain. (2017) 18(9):1027–35. 10.1016/j.jpain.2017.03.008 28400293 PMC5581265

[172] DosSantos MF Ferreira N Toback RL Carvalho AC DaSilva AF. Potential mechanisms supporting the value of motor cortex stimulation to treat chronic pain syndromes. Front Neurosci. (2016) 10:18. 10.3389/fnins.2016.00018 26903788 PMC4749700

[173] DosSantos MF Love TM Martikainen IK Nascimento TD Fregni F Cummiford C Immediate effects of tDCS on the μ-opioid system of a chronic pain patient. Front Psychiatry. (2012) 3:93. 10.3389/fpsyt.2012.00093 23130002 PMC3486958

[174] Sobral M Guiomar R Martins V Ganho-Ávila A. Home-based transcranial direct current stimulation in dual active treatments for symptoms of depression and anxiety: a case series. Front Psychiatry. (2022) 13:947435. 10.3389/fpsyt.2022.947435 36276322 PMC9583668

[175] Woodham RD Selvaraj S Lajmi N Hobday H Sheehan G Ghazi-Noori A-R Home-based transcranial direct current stimulation treatment for major depressive disorder: a fully remote phase 2 randomized sham-controlled trial. Nat Med. (2024) 31:87–95. 10.1038/s41591-024-03305-y 39433921 PMC11750699

[176] Antonioni A Baroni A Fregna G Ahmed I Straudi S. The effectiveness of home-based transcranial direct current stimulation on chronic pain: a systematic review and meta-analysis. Digit Health. (2024) 10:20552076241292677. 10.1177/20552076241292677 39600390 PMC11590159

[177] Wurzman R Hamilton RH Pascual-Leone A Fox MD. An open letter concerning do-it-yourself users of transcranial direct current stimulation. Ann Neurol. (2016) 80(1):1–4. 10.1002/ana.24689 27216434 PMC6050584

[178] Carvalho S Leite J Jones F Morse LR Zafonte R Fregni F. Study adherence in a tDCS longitudinal clinical trial with people with spinal cord injury. Spinal Cord. (2018) 56(5):502–8. 10.1038/s41393-017-0023-5 29234136

[179] Delicado-Miralles M Flix-Diez L Gurdiel-Álvarez F Velasco E Galán-Calle M Lerma Lara S. Temporal dynamics of adverse effects across five sessions of transcranial direct current stimulation. Brain Sci. (2024) 14(5):457. 10.3390/brainsci14050457 38790436 PMC11118034

[180] Rahimibarghani S Fateh HR. Long-lasting mood deterioration following transcranial direct current stimulation treatment for fibromyalgia: a case report. Clin Case Rep. (2023) 11(8):e7712. 10.1002/ccr3.7712 37575465 PMC10415587

[181] Lefaucheur JP Antal A Ayache SS Benninger DH Brunelin J Cogiamanian F Evidence-based guidelines on the therapeutic use of transcranial direct current stimulation (tDCS). Clin Neurophysiol. (2017) 128(1):56–92. 10.1016/j.clinph.2016.10.087 27866120

[182] Alves RL Zortea M Serrano PV Laranjeira VDS Tocchetto BF Ramalho L Modulation of neural networks and symptom correlated in fibromyalgia: a randomized double-blind factorial explanatory clinical trial of home-based transcranial direct current stimulation. medRxiv. (2023). 10.1101/2023.07.05.23292267 PMC11560039 39536019

