# Supplementary material for: Clinical, neurophysiological and neurochemical effects of non-invasive electrical brain stimulation in fibromyalgia syndrome—a systematic review and meta-analysis
Source: Front Pain Res (Lausanne). 2025 Aug 1;6:1593746. doi: 10.3389/fpain.2025.1593746 (PMC12354543; doi:10.3389/fpain.2025.1593746)
Supplement: Supplementary file 1 [file Presentation1.pdf]

Appendix

Supplement 1. PICO Question development tool (Schardt et al., 2007)

What is the problem?

Lack of effective treatment options in FMS. Conventional pharmacological and psychological treatment have shown limited benefits. Non-invasive transcranial electrical brain stimulation (tES) can change neuronal excitability and thereby alter the functioning of the central nervous system, producing changes in cognition and behavior in patients with chronic pain. (FMS) is associated with central sensitization. These altered dynamics and plastic changes in the brains of FMS patients are linked to clinical, neurochemical, and neurophysiological changes.

What are the data and sources of information that validate the problem?

- ☐ Safety and risk management concerns: ________
- ● Data: Peer-reviewed studies
- ☐ Financial information: ________
- ☐ Lack of evidence for current practice: ________
- ☐ Quality indicators: ________
- ☐ Practice observations: ________
- ☐ Other: ________

Why is the problem important and relevant? What would happen if it were not addressed?

High prevalence of FMS worldwide, aprox. 3% in US, 2.5% in Europe. About 20% of chronic widespread musculoskeletal Pain is treated with opioids. Although levels of opioid use and abuse in Europe not near those of the US, there is a rising trend to prescribe Opioids to FMS patients in Europe, too. This far, no one single effective treatment for all FMS patients.

What is the current practice?

Primary treatment option is pharmacological in nature.

Is this a background question to establish the state of the evidence on a topic (with no comparison group) or a foreground question to compare specific interventions?

● Background ☐ Foreground

What are the PICO components?

P (patient, population, or problem):

Adult patients (18 years plus) with diagnosis of FMS

I (intervention):

Non-invasive transcranial current stimulation, tDCS, tACS, tRNS.

C (comparison with other interventions if *foreground* question):

Comparison can include all kinds of treatments or no treatment at all.

O (outcomes):

Improvement of clinical, neurophysiological, neuropsychological, or neurochemical symptoms/variables in FMS, especially pain relief, depression, anxiety, sleep, QoL, cognitive functioning

Initial EBP question:

Do adults with FMS who undergo non-invasive electrical current stimulation – improve on pain relief, mood disorders, sleep, QoL and cognitive Functioning?

List possible search terms for each part of the PICO question:

PICO Element	Possible Search Terms
P	Fibromyalgia syndrome; adult population
I	Non-invasive transcranial current stimulation; transcranial direct current stimulation, transcranial alternating current stimulation; transcranial random noise stimulation
C	---
O	Pain; depression; anxiety; sleep; quality of life; cognitive functioning; neurotransmitter; evoked potentials, Neurochemical change; neurophysiological changes; oscillation, functional connectivity, EEG

What are preliminary inclusion and exclusion criteria (e.g., date, population, setting, other)?

Inclusion:	Exclusion:
(1) Peer-reviewed original studies of FMS tES (i.e., longitudinal studies, pilot studies, pilot randomized controlled trials, randomized controlled clinical quasi-experimental trials, single-case/small group designs, and uncontrolled and controlled pre-post-test studies), (2) adult participants, minimum 18 years of age, and a medical diagnosis of FMS, and (3) are written in English.	(1) Review articles or meta-analyses, (2) comments, editorial, letter, or meeting/congress abstract, (3) non-English publications, secondary musculoskeletal pain samples, samples with no medical diagnosis of FMS, and all studies which did not fulfil the inclusion criteria.

What evidence needs to be reviewed? (Check all that apply)

- ● Peer-reviewed publications (from databases such as PubMed, CINAHL, Embase)
- ☐ Standards (regulatory, professional, community)
- ☐ Clinical Practice Guidelines
- ☐ Organizational data (e.g., quality improvement or financial data, local clinical expertise, patient/family preferences)
- ☐ Evidence-based professional organization position statements
- ☐ Consensus studies (e.g., commissioned reports from the National Academy of Medicine, professional organizations, philanthropic foundations)
- ☐ Other ________

Revised EBP question:

(1) Do adults with FMS who undergo non-invasive electrical current stimulation – anodal or cathodal tDCS, tACS, or tRNS - improve on sleep problems, fatigue, quality of life (QoL), depression, anxiety, cognitive performance or pain intensity?
(2) What are the neurophysiological and neurochemical effects, reflected by oscillatory activity and/or functional connectivity, and neurotransmitter levels of anodal and cathodal tDCS, tACS, tRNS in adults with FMS?

What are measures that indicate if the EBP project is successful? (Measures may be structure, process, and/or outcome)

https://www.cvph.org/data/files/Appendix%20B%20Question%20development%20tool%202022.pdf

Supplement 2. Detailed information about source of evidence selection for all databases

For PubMed

The search structure of the function Advanced Search on PubMed consisted of the following, shown for “tDCS” as an example:
PubMed Advanced Search Builder – Search in “All Fields” - Search: (((tDCS) OR (transcranial direct current stimulation))) AND (fibromyalgia) - Saved search Filters: Filters applied: Case Reports, Clinical Study, Clinical Trial, Comparative Study, Controlled Clinical Trial, Evaluation Study, Multicenter Study, Pragmatic Clinical Trial, Randomized Controlled Trial, Humans, English, Adult: 19+ years, from 2013/4/1 - 2023/4/14.
Sort by: Most Recent
(("transcranial direct current stimulation"[MeSH Terms] OR ("transcranial"[All Fields] AND "direct"[All Fields] AND "current"[All Fields] AND "stimulation"[All Fields]) OR "transcranial direct current stimulation"[All Fields] OR "tdcs"[All Fields] OR ("transcranial direct current stimulation"[MeSH Terms] OR ("transcranial"[All Fields] AND "direct"[All Fields] AND "current"[All Fields] AND "stimulation"[All Fields]) OR "transcranial direct current stimulation"[All Fields])) AND ("fibromyalgia"[MeSH Terms] OR "fibromyalgia"[All Fields] OR "fibromyalgias"[All Fields])) AND ((clinicaltrial[Filter] OR randomizedcontrolledtrial[Filter]) AND (humans[Filter]) AND (2013/4/1:2023/4/14[pdat]) AND (english[Filter]) AND (alladult[Filter]))

For Wiley Library Online (Cochrane Controlled Trial Register)

Advanced Search - Search anywhere - Terms & Filters as mentioned above in “Search Strategy”.
transcranial current direct stimulation AND fibromyalgia - Filters 2013-2023 // Journals // English

For opengrey.org, via Data Archiving and Network Services (DANS)

Advanced search – Any field - “fibromyalgia AND transcranial direct current stimulation” / “fibromyalgia AND transcranial alternating current stimulation” / “fibromyalgia AND transcranial random noise stimulation”

For LILACS

Virtual Health Library - Advanced search - Title/abstract/Subject - “fibromyalgia AND transcranial direct current stimulation” - Filters: English language / from 2013 - 2023 - Type of study: Controlled clinical trial / Prognostic study / Risk factors / Diagnostic study / Observational study / Etiology study / Qualitative research

For ClinicalTrials.gov

tDCS OR transcranial direct current stimulation | Fibromyalgia | Adult, Older Adult
tACS OR transcranial alternating current stimulation | Fibromyalgia | Adult, Older Adult
tRNS OR transcranial random noise stimulation | Fibromyalgia | Adult, Older Adult

For Scopus

(fibromyalgia) AND (((brain OR transcrani* OR non-invasive OR noninvasive) AND stimulat*) OR tdcs OR tacs OR trns) AND (LIMIT-TO (OA , "all")) AND (LIMIT-TO (PUBSTAGE , "aip")) AND (LIMIT-TO (LANGUAGE , "English"))

For PsycINFO

(DE "Fibromyalgia") AND ((DE "Transcranial Direct Current Stimulation") OR (Fibromyalgia AND (((brain OR transcrani OR non-invasive OR noninvasive) AND stimulat*) OR tDCS OR tACS OR tRNS Filter for: English language, peer reviewed journal, year range 1913-2023*

Supplement 3. Interrater reliability *(CW and HC)*

Calculation of percentage agreement

number of zeros	1	
number of items	57	
percent agreement	98,24%	
	A	B
Cohen´s kappa = 0.9588	Y = 39	Y = 40
	N = 18	N = 17

Supplement 4. PEDro scale (1999).

PEDro scale

1. eligibility criteria were specified	no ❑ yes ❑ where:
2. subjects were randomly allocated to groups (in a crossover study, subjects were randomly allocated an order in which treatments were received)	no ❑ yes ❑ where:
3. allocation was concealed	no ❑ yes ❑ where:
4. the groups were similar at baseline regarding the most important prognostic indicators	no ❑ yes ❑ where:
5. there was blinding of all subjects	no ❑ yes ❑ where:
6. there was blinding of all therapists who administered the therapy	no ❑ yes ❑ where:
7. there was blinding of all assessors who measured at least one key outcome	no ❑ yes ❑ where:
8. measures of at least one key outcome were obtained from more than 85% of the subjects initially allocated to groups	no ❑ yes ❑ where:
9. all subjects for whom outcome measures were available received the treatment or control condition as allocated or, where this was not the case, data for at least one key outcome was analysed by "intention to treat"	no ❑ yes ❑ where:
10. the results of between-group statistical comparisons are reported for at least one key outcome	no ❑ yes ❑ where:
11. the study provides both point measures and measures of variability for at least one key outcome	no ❑ yes ❑ where:

Supplement 5. Quality assessment standard for cross-over studies (Ding et al., 2015).

Item	Description	Scoring
1. Appropriate cross-over design	Three points are considered: (1) the condition of the patients should be chronic and stable; (2) the intervention should not provide permanent change, but rather temporary relief; (3) the effect of the first intervention should not last into the second treatment period.	Low: all the three points are absolutely correct;
		Unclear: it hard to judge because some information was missing or ambiguous;
		High: one or more points are incorrect.
2. Randomized treatment order	The order of receiving treatments should be randomized adequately.	Low: the method is appropriate and clearly described;
		Unclear: it is described as "randomized", but it is hard to judge whether the implementation was adequate because some information (method, etc.) was not provided;
		High: the method is inappropriate, or no randomization is applied.
3. Carry-over effect	The authors should evaluate the carry-over effect and provide relevant information clearly.	Low: carry-over effect was evaluated and the results showed no carry-over effect;
		Unclear: carry-over effect was not evaluated, and it is hard for evaluators to judge;
		High: carry-over effect was evaluated and the results showed apparent carry-over effect, or indicated evidently from some other provided information.
4. Unbiased data	That only first-period data are available is considered a risk of bias.	Low: data for every period are provided;
		Unclear: data are unavailable for part of outcomes, or only analytical results are provided and it is hard to judge whether the results are analyzed based only on data from the first-period or every period.
		High: only first-period data are available.
5. Allocation concealment	The study should apply appropriate approaches to ensure the allocation sequence is concealed.	Low: allocation sequence was concealed adequately by appropriate methods;
		Unclear: concealment approaches were not described, or relevant information was ambiguous;
		High: no approaches to allocation concealment were used, or concealed inadequately.
6. Blinding	The study should apply a proper blinding method to prevent performance and detection bias. Those involved in blinding (participants, doctors, measurers, or analysts) depends on the particularity of the studies.	Low: appropriate blinding method was applied; No blinding, but the outcome and the outcome measurement are not likely to be influenced by lack of blinding;
		Unclear: relevant information was not provided;
		High: no blinding method was applied, or applied incorrectly, or ineffectively, which very likely affected the outcome.
7. Incomplete outcome data	The authors should provide relevant information about the completeness of outcome data, including the level of incompleteness, reasons, and analytic method to tackle these data shortcomings, etc.	Low: no missing outcome data, or the reason is acceptable, or missing outcome data were appropriate analyzed;
		Unclear: it is hard to judge because some information was not provided;
		High: missing outcome data existed and the reasons were unacceptable, and the analytic method was inappropriate.
8. Selective outcome reporting	The authors should report all the outcomes fully. Selective reporting of part of outcomes or data for an outcome or subsets of the data or analyses using the same data and etc. should be avoided.	Low: fully reported;
		Unclear: it is hard to judge due to the unavailability of some original information;
		High: the reports of the study suggest a high risk of selective outcome reporting.
9. Other bias	Any other potential risk of bias that may affect the quality of cross-over studies.	Low: the study is apparently free of other problems;
		Unclear: whether certain problems existed and led to a risk of bias is uncertain;
		High: high risk of bias existed due to evident problems.

NOTE: the standard was summarized from the Cochrane Collaboration's tool for assessing risk of bias and the Cochrane handbook`s suggestions for assessing risk of bias in cross-over studies. The assessment of some items, especially items 5–8, are almost the same as that described in Cochrane Collaboration's tool for assessing the risk of bias.

doi:10.1371/journal.pone.0120519.t001

Supplement 6. The MINORS ROB tool to assess single-arm trials (Slim et al., 2003).

Slim, K., Nini, E., Forestier, D., Kwiatkowski, F., Panis, Y., & Chipponi, J. (2003). Methodological index for non-randomized studies (minors): development and validation of a new instrument. ANZ journal of surgery, 73(9), 712–716. https://doi.org/10.1046/j.1445-2197.2003.02748.x

Table 2. The revised and validated version of MINORS

Methodological items for non-randomized studies	Score†
1. **A clearly stated aim:** the question addressed should be precise and relevant in the light of available literature	
2. **Inclusion of consecutive patients**: all patients potentially fit for inclusion (satisfying the criteria for inclusion) have been included in the study during the study period (no exclusion or details about the reasons for exclusion)	
3. **Prospective collection of data**: data were collected according to a protocol established before the beginning of the study	
4. **Endpoints appropriate to the aim of the study**: unambiguous explanation of the criteria used to evaluate the main outcome which should be in accordance with the question addressed by the study. Also, the endpoints should be assessed on an intention-to-treat basis.	
5. **Unbiased assessment of the study endpoint**: blind evaluation of objective endpoints and double-blind evaluation of subjective endpoints. Otherwise the reasons for not blinding should be stated	
6. **Follow-up period appropriate to the aim of the study**: the follow-up should be sufficiently long to allow the assessment of the main endpoint and possible adverse events	
7. **Loss to follow up less than 5%**: all patients should be included in the follow up. Otherwise, the proportion lost to follow up should not exceed the proportion experiencing the major endpoint	
8. **Prospective calculation of the study size**: information of the size of detectable difference of interest with a calculation of 95% confidence interval, according to the expected incidence of the outcome event, and information about the level for statistical significance and estimates of power when comparing the outcomes	
Additional criteria in the case of comparative study	
9. **An adequate control group**: having a gold standard diagnostic test or therapeutic intervention recognized as the optimal intervention according to the available published data	
10. **Contemporary groups**: control and studied group should be managed during the same time period (no historical comparison)	
11. **Baseline equivalence of groups**: the groups should be similar regarding the criteria other than the studied endpoints. Absence of confounding factors that could bias the interpretation of the results	
12. **Adequate statistical analyses**: whether the statistics were in accordance with the type of study with calculation of confidence intervals or relative risk	

†The items are scored 0 (not reported), 1 (reported but inadequate) or 2 (reported and adequate). The global ideal score being 16 for non-comparative studies and 24 for comparative studies.

Supplement 7. Quantitative Data Synthesis

All analyses were performed manually using Comprehensive Meta-Analysis Software, version 4 (www.meta-analysis.com). Effect sizes for continuous outcomes - including pain intensity, quality of life, depression, pressure pain threshold, heat pain threshold, and heat pain tolerance - were calculated using Hedges' g with 95% confidence intervals. Hedges' g, a variation of Cohen's d, was chosen to correct for bias associated with the small sample sizes typical of the included studies (Lakens, 2013).

To assess the efficacy of active tDCS, one study was selected as a reference group. This reference study served as a baseline for comparing the effects of anodal tDCS across other studies. For each study, Hedges' g was computed by comparing its post-treatment mean to that of the reference study. These effect sizes were then pooled to generate an overall estimate of anodal tDCS efficacy within the subgroup.

Selecting a single reference study allowed for standardized comparisons across studies in the subgroup. The chosen reference study was representative, matched other studies in participant characteristics, intervention protocols, and outcome measures, and exhibited a low risk of bias. Furthermore, sensitivity analyses were conducted to evaluate the robustness of the findings relative to the chosen reference.

Hedges' g was calculated using the following formula:

$$g = \frac{M_i - M_{\text{ref}}}{SD_{\text{pooled}}}$$

Where M_i is the mean outcome score post-treatment, M_{ref} refers to the mean outcome score post-treatment in the reference group, n is the sample size, SD_i is the standard deviation of the outcome in question post-treatment, SD_{ref} is the standard deviation of the outcome in the reference group, n_i refers to the sample size of the study and n_{ref} refers to the sample size of the reference group.

Pooled standard deviation was calculated using the formula:

$$SD_{\text{pooled}} = \sqrt{\frac{(n_i - 1) \cdot SD_i^2 + (n_{\text{ref}} - 1) \cdot SD_{\text{ref}}^2}{n_i + n_{\text{ref}} - 2}}$$

Methods

Given the heterogeneity of comparators across included trials, a standardized approach was used in the quantitative synthesis to facilitate indirect comparisons. Specifically, effect sizes (Hedges' *g*) were calculated for each study relative to a selected reference study with low risk of bias and comparable participant and intervention characteristics. This strategy is conceptually aligned with the method described by Edwards et al. (2009) as "indirect comparison using a single common comparator," which they identify as a statistically valid approach for estimating relative effects in the absence of direct comparisons. Our method also follows the rationale of anchored indirect comparisons, as outlined in Bucher et al. (1997), which preserve randomization by comparing treatment effects through a shared comparator arm. Furthermore, the 2024 *Methodological Guideline for Quantitative Evidence Synthesis*(2024) supports anchored indirect comparison methods using aggregate data in connected networks. In line with this, Hemmelmann et al. (2025) describe a related anchor-based framework for estimating treatment effects in contexts lacking direct comparison trials. While our method does not constitute a formal network meta-analysis, it was chosen to ensure consistency in effect estimation across trials with varying control conditions.

Assessment of statistical heterogeneity: variation in intervention effects /results

The following variables have been retrieved for meta-analysis: pain intensity measured by the VAS or the NRS, health status and quality of life assessed by the Fibromyalgia Impact Questionnaire, depression evaluated by the BDI-II, pain pressure threshold, heat pain threshold, and heat pain tolerance.

Effect size measures

To test the null hypothesis, we assumed that the mean effect was zero, that there is no common effect size between the studies (random–effects model).

Pain intensity

For RCTs data from 12 studies comparing post-treatment outcomes of anodal tDCS against sham tDCS or a control group on pain intensity including 445 participants could be analyzed and resulted in a significant overall effect in favor of the a-tDCS condition using random-effects model ($Z = 3.01$, p 0.05; SMD = -0.63, 95%CI -1.03, -0.22). I^2 statistics suggested substantial heterogeneity ($I^2 = 75\%$) across the studies (Deeks et al., 2020; Dettori et al., 2021). As it was for the cross-over trials, two studies with a total of 60 participants showed no significant overall effect between a-tDCS and sham or control intervention on pain intensity ($Z = 0.97$, $p > 0.05$; SMD = -0.25, 95%CI -0.76, -0.26). To summarize, anodal tDCS in participants with FMS had a significant effect on pain levels as compared to sham or control conditions. Pain levels decreased after a-tDCS in FMS patients in the RCTs, no such result was found for the cross-over trials. In regard of the

practical significance of the results, we calculated the overall effect size using Hedges´ g. First, we determined the effect sizes – Hedges´ g - for each group (group 2 to 12) relative to the reference group. To pool the effect sizes obtained for each group relative to the refence group, we used DerSimonian-and-Laird random effects model, which provides a more conservative estimate of the overall effect size (Borenstein et al., 2011; DerSimonian & Laird, 1986):

$ES_{overall} = \sum^{n}_{i=1} w_i x\, g_i / \sum^{n}_{i=1} w_i$

$\text{Variance}_{\text{overall}} = 1 / \sum^{n}_{i=1} w_i$

Confidence Interval $(CI) = ES_{overall}\ \ Z\ \text{Variance}_{\text{overall}}$

For pain intensity, the overall effect size was 0.00186 with a 95% CI for the overall effect size of 0.00186 0.20522, which translates to an interval of –0.20336 to 0.20708 (Table 8). In summary, the effect size of 0.00186 indicates a very small effect of anodal tDCS on pain intensity across the analyzed studies (Cohen, 1988; Lakens, 2013). Due to substantial heterogeneity of the included studies and the variability of the risk of bias, we conducted a sensitivity analysis to enhance the transparency and robustness of our findings. We excluded all studies which were not low regarding risk of bias (de Melo et al., 2020; Fagerlund et al., 2015; Khedr et al., 2017; Matias, Cavalcante et al., 2022; Matias, Germano et al., 2022; Mendonca et al., 2016; Paula et al., 2022) but this exclusion did not significantly affect the overall findings regarding the efficacy of anodal tDCS in the reduction of pain intensity. We obtained an effect size close to –0.28, while statistically significant, it may have limited practical significance in the context of treatment efficacy. In practical terms, the observed difference in pain intensity between anodal tDCS and sham tDCS may not be large enough to warrant clinical intervention or to produce meaningful improvements in patients´ outcomes. It is to bear in mind that, in the presence of significant heterogeneity as it is the case here, pooling effect sizes may not accurately represent the true treatment effect, so the interpretation of the pooled effect size remains challenging.

Table 7. Pain intensity (VAS/NRS) post intervention for between subjects and within subjects cross-over designs

	a-tDCS			sham / other				Std. mean difference	Risk of Bias					
**Study or Subgroup**	**Mean**	**SD**	**Total**	**Mean**	**SD**	**Total**	**Weight**	**IV, Random, 95% CI**	**A**	**B**	**C**	**D**	**E**	**F**
**1.1.1 Pain intensity POST intervention between subjects design**														
Arroyo-Fernández et al.	5.07	2.05	40	5.08	2.06	40	9.0%	-0.00 [-0.44 , 0.43]	+	+	+	+	+	+
Brietzke et al.	2.75	0.85	10	5.33	0.81	10	4.1%	-2.98 [-4.33 , -1.62]	+	+	+	+	+	+
de Melo et al.	5.05	2.49	11	4.82	3.34	11	6.5%	0.08 [-0.76 , 0.91]	+	+	−	+	+	?
Fagerlund et al.	4.26	1.9	24	5.22	1.5	24	8.1%	-0.55 [-1.13 , 0.03]	+	+	?	?	+	?
Forogh et al.	5.93	2.15	15	4.87	2.2	15	7.2%	0.47 [-0.25 , 1.20]	+	+	+	+	+	+
Jales Junior et al.	3.6	1.84	10	5.6	2.51	10	6.0%	-0.87 [-1.80 , 0.06]	+	+	+	+	+	+
Khedr et al.	5.8	1.1	20	7.2	0.9	20	7.4%	-1.37 [-2.06 , -0.67]	+	+	−	+	+	?
Loreti et al.	3.29	1.39	17	5.28	1.18	18	7.0%	-1.51 [-2.27 , -0.75]	+	+	+	+	+	+
Matias, Cavalcante et al.	4.93	2.31	15	5.66	2.66	15	7.2%	-0.29 [-1.00 , 0.43]	+	+	+	?	+	?
Matias, Germano et al.	4.99	2.75	23	5.22	3.45	24	8.2%	-0.07 [-0.64 , 0.50]	+	+	+	?	+	?
Mendonca et al.	5.7	2.31	15	6.8	2	15	7.2%	-0.50 [-1.22 , 0.23]	+	+	+	?	+	?
Paula et al.	4.41	0.55	22	5	0.59	21	7.7%	-1.02 [-1.66 , -0.38]	+	+	+	?	+	?
**Subtotal (95% CI)**			**222**			**223**	**85.6%**	**-0.63 [-1.03 , -0.22]**						
Heterogeneity: Tau² = 0.37; Chi² = 44.86, df = 11 (P < 0.00001); I² = 75%														
Test for overall effect: Z = 3.01 (P = 0.003)														
**1.1.2 Pain intensity POST intervention crossover within subjects design**														
Foerster et al.	3.3	2.8	12	4.1	2.1	12	6.7%	-0.31 [-1.12 , 0.49]	−	+	−	+	+	−
Villamar et al.	4.07	1.61	18	4.41	1.52	18	7.6%	-0.21 [-0.87 , 0.44]	+	+	+	?	+	?
**Subtotal (95% CI)**			**30**			**30**	**14.4%**	**-0.25 [-0.76 , 0.26]**						
Heterogeneity: Tau² = 0.00; Chi² = 0.04, df = 1 (P = 0.85); I² = 0%														
Test for overall effect: Z = 0.97 (P = 0.33)														
**Total (95% CI)**			**252**			**253**	**100.0%**	**-0.57 [-0.92 , -0.21]**						
Heterogeneity: Tau² = 0.31; Chi² = 45.64, df = 13 (P < 0.0001); I² = 72%														
Test for overall effect: Z = 3.15 (P = 0.002)														
Test for subgroup differences: Chi² = 1.26, df = 1 (P = 0.26), I² = 20.8%														

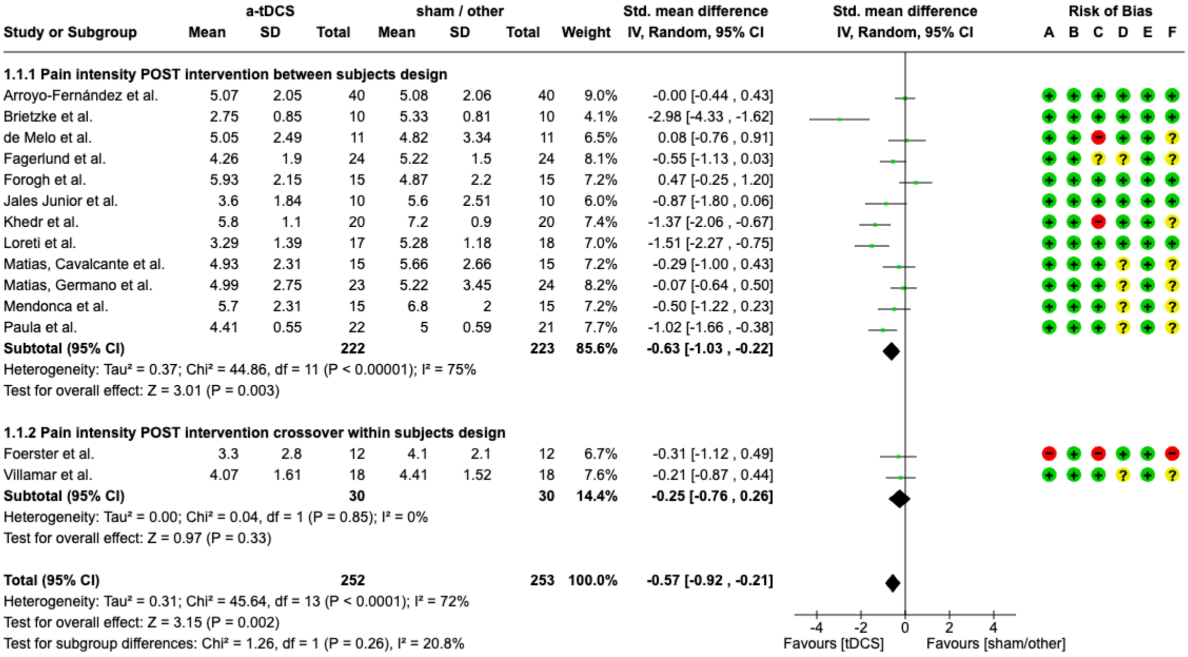

Risk of bias legend
(A) Bias arising from the randomization process
(B) Bias due to deviations from intended interventions
(C) Bias due to missing outcome data
(D) Bias in measurement of the outcome
(E) Bias in selection of the reported result
(F) Overall bias

Table 8. Quality of life (FIQ) post intervention between subjects design

	a-tDCS			sham / other				Std. mean difference	Risk of Bias					
**Study or Subgroup**	**Mean**	**SD**	**Total**	**Mean**	**SD**	**Total**	**Weight**	**IV, Random, 95% CI**	**A**	**B**	**C**	**D**	**E**	**F**
Arroyo-Fernández et al.	54.87	18.38	40	54.1	20.21	40	13.3%	0.04 [-0.40 , 0.48]	+	+	+	+	+	+
Fagerlund et al.	41.51	23.99	24	47.27	18.16	24	12.0%	-0.27 [-0.83 , 0.30]	+	+	?	?	+	?
Jales Junior et al.	35.42	12.841	10	42.42	28.241	10	8.8%	-0.31 [-1.19 , 0.58]	+	+	+	+	+	+
Loreti et al.	31.92	3.503	17	41.54	3.986	18	8.6%	-2.50 [-3.41 , -1.59]	+	+	+	+	+	+
Matias, Cavalcante et al.	60.83	14.57	15	58.72	16.84	15	10.4%	0.13 [-0.59 , 0.85]	+	+	+	?	+	?
Matias, Germano et al.	53.56	18.83	23	58.94	16.03	24	11.9%	-0.30 [-0.88 , 0.27]	+	+	+	?	+	?
Samartin-Veiga, Gonzalez-Villar et al. over DLPFC	50.73	22.14	26	53.75	23.3	25	12.2%	-0.13 [-0.68 , 0.42]	+	+	+	+	+	+
Samartin-Veiga, Gonzalez-Villar et al. over M1	56.27	23.68	29	53.75	23.3	25	12.3%	0.11 [-0.43 , 0.64]	+	+	+	+	+	+
Serrano et al.	58.42	15.53	24	64.73	10.94	12	10.6%	-0.43 [-1.14 , 0.27]	+	+	+	+	+	+
**Total (95% CI)**			**208**			**193**	**100.0%**	**-0.34 [-0.73 , 0.05]**						
Heterogeneity: Tau² = 0.25; Chi² = 28.46, df = 8 (P = 0.0004); I² = 72%														
Test for overall effect: Z = 1.70 (P = 0.09)														
Test for subgroup differences: Not applicable														

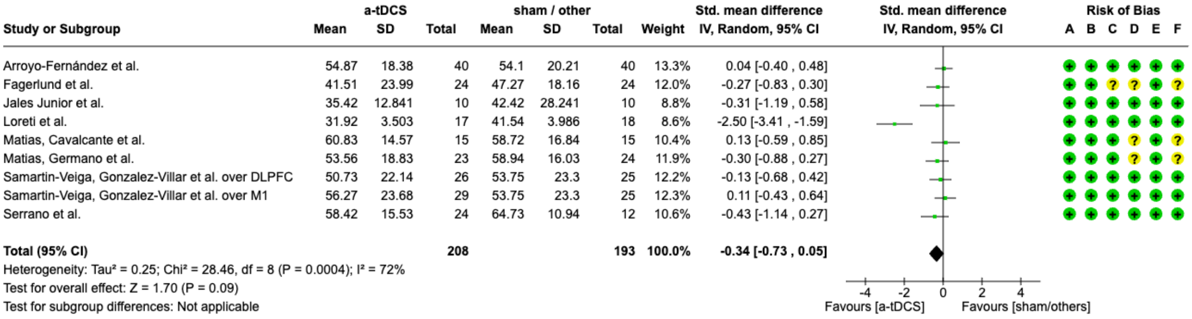

Risk of bias legend
(A) Bias arising from the randomization process
(B) Bias due to deviations from intended interventions
(C) Bias due to missing outcome data
(D) Bias in measurement of the outcome
(E) Bias in selection of the reported result
(F) Overall bias

For RCTs data from 9 studies comparing post-treatment outcomes of anodal tDCS against sham tDCS or a control group on QoL including 208 participants could be analyzed and no significant overall effect in favor of the a-tDCS condition using random-effects model could be find (Z = 1.70, p = 0.09; SMD = -0.34, 95%CI –0.73, 0.05) (Table 9). In conclusion, the effect of anodal tDCS on quality of life does not appear to be statistically significant, and the observed effect size is relatively small and uncertain.

Table 9. Depression (BDI-II) post intervention between subjects design

Study or Subgroup	a-tDCS Mean	a-tDCS SD	a-tDCS Total	sham/others Mean	sham/others SD	sham/others Total	Weight	Std. mean difference IV, Random, 95% CI	A	B	C	D	E	F
Arroyo-Fernández et al.	17.25	8.33	40	15.9	9.09	40	24.2%	0.15 [-0.29 , 0.59]	+	+	+	+	+	+
Brietzke et al.	11.8	5.63	10	21.5	6.6	10	14.2%	-1.51 [-2.54 , -0.49]	+	+	+	+	+	+
Caumo et al.	13.43	7.75	32	18.77	9.97	16	21.0%	-0.62 [-1.23 , -0.00]	+	+	+	+	+	+
Matias, Cavalcante et al.	15.29	9.83	23	15.64	11.49	24	21.8%	-0.03 [-0.60 , 0.54]	+	+	+	?	+	?
Mendonca et al.	20.2	3	15	18.5	3.2	15	18.9%	0.53 [-0.20 , 1.26]	+	+	+	?	+	?
**Total (95% CI)**			**120**			**105**	**100.0%**	**-0.21 [-0.75 , 0.33]**						

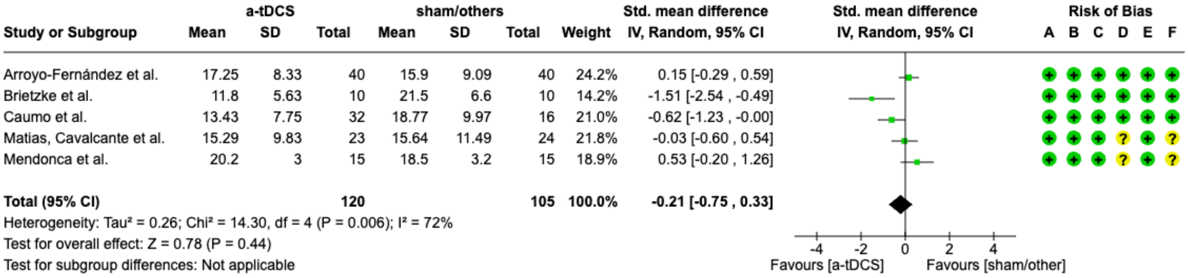

Heterogeneity: Tau² = 0.26; Chi² = 14.30, df = 4 (P = 0.006); I² = 72%
Test for overall effect: Z = 0.78 (P = 0.44)
Test for subgroup differences: Not applicable

Risk of bias legend
(A) Bias arising from the randomization process
(B) Bias due to deviations from intended interventions
(C) Bias due to missing outcome data
(D) Bias in measurement of the outcome
(E) Bias in selection of the reported result
(F) Overall bias

For RCTs data from 5 studies comparing post-treatment outcomes of anodal tDCS against sham tDCS or a control group on depression including 120 participants could be analyzed and no significant overall effect in favor of the a-tDCS condition using random-effects model could be find (Z = 0.78, p 0.44; SMD = -0.21, 95%CI –0.75, 0.33) (Table 10). The overall effect suggests that there was no statistically significant overall effect of anodal tDCS on depression in patients with FMS. On average, anodal tDCS does not appear to improve depression symptoms in this population. The significant heterogeneity and the high I^2 = 72% suggest substantial variability among the effect sizes of the included studies. While anodal tDCS shows promise as a potential treatment for depressive symptoms in patients with FMS, the evidence from meta-analysis does not support a significant overall effect. The presence of heterogeneity also suggests that there are other factors influencing the treatment effect across studies, which need to be further explored. Further research with larger sample sizes and more homogeneous study designs are necessary to draw more definitive conclusions about the effectiveness of anodal tDCS for depression in this population.

Table 10. Pain pressure threshold (PPT in kgf) post intervention between subjects design*

Study or Subgroup	a-tDCS Mean	a-tDCS SD	a-tDCS Total	sham/others Mean	sham/others SD	sham/others Total	Weight	Std. mean difference IV, Random, 95% CI	A	B	C	D	E	F
Brietzke et al.	4.14	0.73	10	3.02	0.72	10	18.2%	1.48 [0.46 , 2.49]	+	+	+	+	+	+
Khedr et al.	342.2	66.2	20	214.3	33.8	20	19.4%	2.39 [1.56 , 3.21]	+	+	-	+	+	?
Matias, Cavalcante et al.	1.64	0.6	15	1.79	0.95	15	20.2%	-0.18 [-0.90 , 0.53]	+	+	+	?	+	?
Samartin-Veiga, Pidal-Miranda et al. over DLPFC	2	0.97	27	2.31	1.12	25	21.1%	-0.29 [-0.84 , 0.25]	+	+	+	+	+	+
Samartin-Veiga, Pidal-Miranda et al. over M1	2	1.26	27	2.31	1.12	25	21.1%	-0.26 [-0.80 , 0.29]	+	+	+	+	+	+
**Total (95% CI)**			**99**			**95**	**100.0%**	**0.58 [-0.40 , 1.56]**						

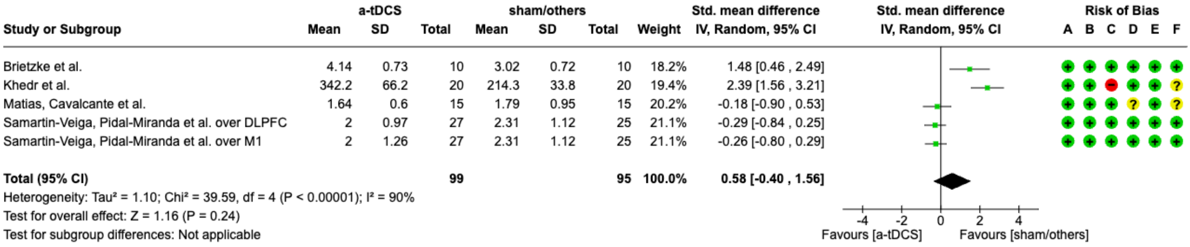

Heterogeneity: Tau² = 1.10; Chi² = 39.59, df = 4 (P < 0.00001); I² = 90%
Test for overall effect: Z = 1.16 (P = 0.24)
Test for subgroup differences: Not applicable

Risk of bias legend
(A) Bias arising from the randomization process
(B) Bias due to deviations from intended interventions
(C) Bias due to missing outcome data
(D) Bias in measurement of the outcome
(E) Bias in selection of the reported result
(F) Overall bias

*In this review, pain pressure threshold (PPT) values are reported in kilogram-force (kgf) as originally presented in the included studies. We acknowledge that kgf is a non-SI unit of force, with 1 kgf approximately equal to 9.8 newtons (N), the standard SI unit. To maintain consistency with source data and avoid introducing conversion errors, PPT values were not converted to newtons.

For RCTs data from 5 studies comparing post-treatment outcomes of anodal tDCS against sham tDCS or a control group on PPT including 99 participants could be analyzed and no significant overall effect in favor of the a-tDCS condition using random-effects model could be find (Z = 1.16, p = 0.24; SMD = 0.58, 95%CI –0.40, 1.56) (Table 11). The test for overall effect suggests that there is no statistically significant overall effect of anodal tDCS on PPT in patients with FMS. On average, anodal tDCS did not appear to improve the PPT in this population. The significant heterogeneity and the high I^2 = 90% suggest substantial variability among the effect sizes of the

included studies. The presence of heterogeneity suggests that there are other factors influencing the PPT across studies, which need to be further explored. Further research with larger sample sizes and more homogeneous study designs are necessary to draw more definitive conclusions about the effectiveness of anodal tDCS for improving the PPT in this population.

Table 11. Heat pain threshold (HPTh in C°) post intervention between subjects design

Study or Subgroup	a-tDCS Mean	a-tDCS SD	a-tDCS Total	sham/others Mean	sham/others SD	sham/others Total	Weight	Std. mean difference IV, Random, 95% CI	Risk of Bias A	B	C	D	E	F
Brietzke et al.	38.86	2.39	10	35.86	1.92	10	40.8%	1.33 [0.34 , 2.31]	+	+	+	+	+	+
Caumo et al.	37.38	2.56	32	36.37	2.4	16	59.2%	0.40 [-0.21 , 1.00]	+	+	+	+	+	+
**Total (95% CI)**			**42**			**26**	**100.0%**	**0.78 [-0.12 , 1.67]**						

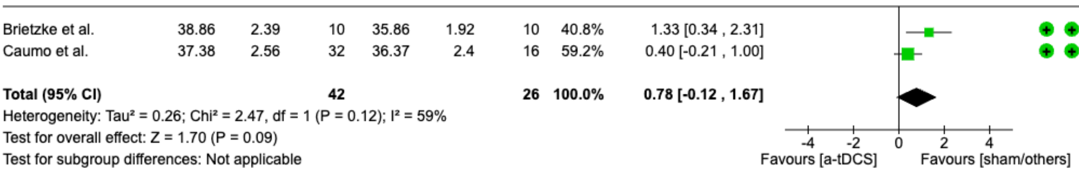

Heterogeneity: Tau² = 0.26; Chi² = 2.47, df = 1 (P = 0.12); I² = 59%
Test for overall effect: Z = 1.70 (P = 0.09)
Test for subgroup differences: Not applicable

Risk of bias legend
(A) Bias arising from the randomization process
(B) Bias due to deviations from intended interventions
(C) Bias due to missing outcome data
(D) Bias in measurement of the outcome
(E) Bias in selection of the reported result
(F) Overall bias

For RCTs data from 2 studies comparing post-treatment outcomes of anodal tDCS against sham tDCS or a control group on HPTh including 42 participants could be analyzed and no significant overall effect in favor of the a-tDCS condition using random-effects model could be find (Z = 1.70, p = 0.09; SMD = 0.78, 95%CI –0.12, 1.67) (Table 12). The test for overall effect suggests that there is no statistically significant overall effect of anodal tDCS on HPTh in patients with FMS. On average, anodal tDCS does not appear to improve the HPTh in this population. The substantial heterogeneity of I^2 = 59% suggests substantial variability among the effect sizes of the included studies. The presence of heterogeneity suggests that there are other factors influencing the HPTh across studies, which need to be further explored. Further research with larger sample sizes and more homogeneous study designs is necessary to draw more definitive conclusions about the effectiveness of anodal tDCS for improving the HPTh in this population.

Table 12. Heat pain threshold (HPTh in C°) post intervention crossover within subjects design

Study or Subgroup	a-tDCS Mean	a-tDCS SD	a-tDCS Total	sham Mean	sham SD	sham Total	Weight	Std. mean difference IV, Random, 95% CI	Risk of Bias A	B	C	D	E	F
Schein et al.	37.75	2.35	15	37.97	3.21	16	48.4%	-0.08 [-0.78 , 0.63]	+	+	+	?	+	?
Silva et al.	40.81	2.86	20	38.88	2.16	20	51.6%	0.75 [0.10 , 1.39]	+	+	+	?	+	?
**Total (95% CI)**			**35**			**36**	**100.0%**	**0.35 [-0.46 , 1.15]**						

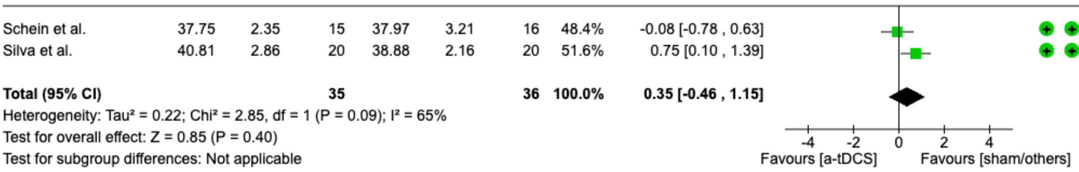

Heterogeneity: Tau² = 0.22; Chi² = 2.85, df = 1 (P = 0.09); I² = 65%
Test for overall effect: Z = 0.85 (P = 0.40)
Test for subgroup differences: Not applicable

Risk of bias legend
(A) Bias arising from the randomization process
(B) Bias due to deviations from intended interventions
(C) Bias due to missing outcome data
(D) Bias in measurement of the outcome
(E) Bias in selection of the reported result
(F) Overall bias

For within-subjects cross-over design data from 2 studies comparing post-treatment outcomes of anodal tDCS against sham tDCS or a control group on HPTh including 35 participants could be analyzed and no significant overall effect in favor of the a-tDCS condition using random-effects model could be find (Z = 0.85, p = 0.40; SMD = 0.35, 95%CI –0.46, 1.15) (Table 13). As with the earlier mentioned between-subjects designs, the test for overall effect indicates that there is no statistically significant overall effect of anodal tDCS on HPTh in patients with FMS. On average,

anodal tDCS does not appear to improve the HPTh in this population. The substantial heterogeneity of $I^2 = 65\%$ shows substantial variability among the effect sizes of the two studies.

Table 13. Heat pain tolerance (HPTo in C°) post intervention between subjects design

	a-tDCS			sham/others				Std. mean difference	Risk of Bias					
Study or Subgroup	Mean	SD	Total	Mean	SD	Total	Weight	IV, Random, 95% CI	A	B	C	D	E	F
Brietzke et al.	45.27	2.52	10	42.89	3.32	10	39.5%	0.77 [-0.14 , 1.69]	+	+	+	+	+	+
Caumo et al.	44.69	2.59	32	44.68	2.27	16	60.5%	0.00 [-0.60 , 0.60]	+	+	+	+	+	+
**Total (95% CI)**			**42**			**26**	**100.0%**	**0.31 [-0.43 , 1.04]**						

Heterogeneity: Tau² = 0.14; Chi² = 1.90, df = 1 (P = 0.17); I² = 47%
Test for overall effect: Z = 0.82 (P = 0.41)
Test for subgroup differences: Not applicable

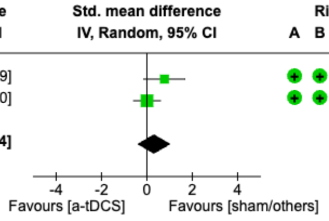

Risk of bias legend
(A) Bias arising from the randomization process
(B) Bias due to deviations from intended interventions
(C) Bias due to missing outcome data
(D) Bias in measurement of the outcome
(E) Bias in selection of the reported result
(F) Overall bias

For RCTs data from 2 studies comparing post-treatment outcomes of anodal tDCS against sham tDCS or a control group on HPTo including 42 participants could be analyzed and no significant overall effect in favor of the a-tDCS condition using random-effects model could be find ($Z = 0.82$, $p = 0.41$; SMD = 0.31, 95%CI –0.43, 1.04) (Table 13). The test for overall effect shows that there is no statistically significant overall effect of anodal tDCS on HPTo in patients with FMS. On average, anodal tDCS did not appear to improve the HPTo in this population. The heterogeneity of $I^2 = 47\%$ suggests moderate variability among the effect sizes of the two studies, which is lower as seen in the former analyses, It can be concluded that approximately half of the variability in effect sizes is due to factors other than random sampling error within those two studies. While moderate heterogeneity is not as extreme as substantial or considerable heterogeneity, it still must be interpreted with caution.

Evaluation of the meta-analysis outcomes

Although the meta-analysis did not quantitatively evaluate the influence of treatment duration, number of sessions, and stimulation location, these variables are likely to play a critical role in the efficacy of anodal tDCS. For example, stimulation of the M1 has been suggested to modulate pain processing through its connections with the thalamus and other pain-related brain regions (DosSantos et al., 2016), which may explain its frequent preference over other targets such as the DLPFC. Additionally, treatment intensity and duration can influence neuroplastic changes and clinical outcomes, with longer or repeated sessions potentially yielding more sustained effects. Future studies should systematically investigate these parameters to clarify their impact and optimize tDCS protocols for fibromyalgia.

Evaluating the practical significance of anodal tDCS effects in FMS, we were able to calculate effect sizes for six outcomes: pain intensity, quality of life, depression, pain pressure threshold, heat pain threshold, and heat pain tolerance. Among these, only pain intensity showed a statistically significant effect; however, the overall effect size was negligible (Hedges' $g \approx 0.002$) and remained small (approaching –0.28) even after sensitivity analyses excluding high-risk studies.

This suggests that while detectable, the effect may not be clinically meaningful. For the other outcomes - quality of life, depression, pain pressure threshold, heat pain threshold, and heat pain tolerance - no significant effects were observed. Substantial heterogeneity and methodological variability across studies further complicate interpretation and limit the generalizability of the findings. These results highlight the need for larger, better-controlled trials and underscore the importance of incorporating clinically relevant thresholds to more accurately determine the utility of anodal tDCS in fibromyalgia treatment.

Limitations of the Meta-Analysis

While the quantitative synthesis was methodologically anchored to a selected reference study to facilitate standardization, we acknowledge the limitations inherent in using indirect comparison approaches. As described by Bucher et al. (1997) and Edwards et al. (2009), such methods rely on assumptions of similarity and consistency across studies, and violations of these assumptions may introduce bias. The 2024 *Methodological Guideline for Quantitative Evidence Synthesis* emphasizes the need for exchangeability and homogeneity in the use of anchored indirect comparisons with aggregate data.

Although we selected a reference study that was comparable in terms of participant characteristics, intervention protocol, and methodological quality, and conducted sensitivity analyses to assess robustness, the approach does not fully substitute for head-to-head or network meta-analytic models. The anchor-based indirect comparison model proposed by Hemmelmann et al. (2025) further supports the conceptual validity of this strategy in complex clinical contexts, though it also highlights the need for caution in interpretation when heterogeneity is present.
